# STEAK: A specific tool for transposable elements and retrovirus detection in high-throughput sequencing data

**DOI:** 10.1093/ve/vex023

**Published:** 2017-08-21

**Authors:** Cindy G. Santander, Philippe Gambron, Emanuele Marchi, Timokratis Karamitros, Aris Katzourakis, Gkikas Magiorkinis

**Affiliations:** 1 Department of Zoology, University of Oxford, Oxfordshire, UK; 2 Science and Technology Facilities Council, Rutherford Appleton Laboratory, Harwell Science and Innovation Campus, Didcot, Oxfordshire, UK; 3 Nuffield Department of Medicine, University of Oxford, Oxfordshire, UK; 4 Department of Hygiene, Epidemiology and Medical Statistics, Medical School, National and Kapodistrian University of Athens, Athens, Greece

**Keywords:** endogenous retroviruses, evolution, transposons, HTS, virus integration, mobile element

## Abstract

The advancements of high-throughput genomics have unveiled much about the human genome highlighting the importance of variations between individuals and their contribution to disease. Even though numerous software have been developed to make sense of large genomics datasets, a major short falling of these has been the inability to cope with repetitive regions, specifically to validate structural variants and accordingly assess their role in disease. Here we describe our program *STEAK*, a massively parallel software designed to detect chimeric reads in high-throughput sequencing data for a broad number of applications such as identifying presence/absence, as well as discovery of transposable elements (TEs), and retroviral integrations. We highlight the capabilities of *STEAK* by comparing its efficacy in locating HERV-K HML-2 in clinical whole genome projects, target enrichment sequences, and in the 1000 Genomes CEU Trio to the performance of other TE and virus detecting tools. We show that *STEAK* outperforms other software in terms of computational efficiency, sensitivity, and specificity. We demonstrate that *STEAK* is a robust tool, which allows analysts to flexibly detect and evaluate TE and retroviral integrations in a diverse range of sequencing projects for both research and clinical purposes.

## 1. Background

High-throughput sequencing (HTS) has undoubtedly revolutionised genome sequencing with technology that has seen a 50,000-fold cost drop and an increase in capacity since the days of the Human Genome Project (Liu et al. 2012; Goodwin et al. 2016). Several consortiums, like the Cancer Genome Atlas (TCGA), have all made use of HTS providing both researchers and clinicians with copious amounts of genomic data in the last decade. Most health- and disease-related researches have focused on exons, conventionally considered the ‘functional’ portions of the genome. This has promoted the widespread use of HTS amongst disease consortiums where short-reads constitute a lesser challenge for correct alignment and genome assembly. While short-read lengths remain useful and informative for unique and complex areas of the genome (e.g. the exome), repetitive, or low-complexity regions, suffer from assembly ambiguities that arise due to HTS read length. Even with the recent advances in sequencing technology and with advanced bioinformatics solutions, repetitive regions remain a challenge—partially because of the nature of our current technology (Treangen and Salzberg 2012) and partially because of the strong focus on working with protein-coding parts of the genome.

At least 55% of the human genome is composed of repetitive elements (Lander et al. 2001), mostly transposable elements (TEs). A number of TEs, such as LINEs, SINEs, and SINE-VNTR-Alu (SVAs), have been found to actively move around the human genome with a potential pathological burden (Ostertag et al. 2003; Mills et al. 2007; Solyom and Kazazian 2012; Evrony et al. 2015). HERV-K HML-2 (or HK2) is a thirty-million year-old family of endogenous retroviruses that continued integrating in the human genome even after the human–chimp divergence. Some HK2 integrations remain unfixed in the population (Marchi et al. 2014; Wildschutte et al. 2016), moreover, every individual carries approximately ten polymorphic HK2 integrations (Marchi et al. 2014). The influence that TEs and human endogenous retroviruses (HERVs) have on altering genetic activity due to somatic rearrangements also implies a potential role in the development of disease (Hohn et al. 2013) for example through insertional mutagenesis (Djebali et al. 2012; Solyom et al. 2012; Shukla et al. 2013; Criscione et al. 2014).

Many cohorts have made use of short-read technology, however, the limitations that HTS short-reads pose for studying TEs with disease consortium data now presents an algorithmic and theoretical challenge for mapping reads with repetitive stretches in their original genomic location (Simola and Kim 2011; Li et al. 2012). Most of the available software make use of paired-end read information or chimeric reads (i.e. reads which are part host and part TE), to identify the genomic location of a TE (Keane et al. 2013; Wu et al. 2014). There are several approaches that use similar methods for discovery of TE integrations in comparison to the reference genome (Lee et al. 2012; Keane et al. 2013; Wu et al. 2014). Other algorithms include trimming chimeric reads of the TE portion to then be remapped to the host reference genome (Marchi et al. 2014). Alternatively, some software search for structural variation differences between the reference and HTS data such as insertions, deletions, inversions, inter-, and intra-chromosomal translocations (Chen et al. 2009).

Here we present a broadly applicable approach to annotate (i.e. mark presence or absence) known and characterise unknown insertion sites for TEs in a variety of sequencing projects. We use HK2 as a mobile element model to evaluate the identification of polymorphic integrations because of its standing as an endogenous retrovirus (Boeke and Stoye 1997).

We have generalised the algorithm previously described by Marchi et al. (2014) to develop a program that will assist in marking presence or absence of any given sequence element within a reference genome as well as identify novel integrations of that sequence element compared to the reference genome. We benchmark the ability of our program **S**pecific **T**ransposable **E**lement **A**ligner **(**HERV-**K)** (STEAK) to discover novel TE and exogenous retrovirus insertions in addition to marking the presence/absence of TEs annotated in the reference genome. We evaluate our method on simulated data as well as high-coverage HTS projects, such as those used in clinical WGS, and compare to competitive systems (Table 1).

**Table 1.** Software for detecting TEs and viruses in WGS data.

Software	Detection target	Detection method	Detects in reference?	Requires specific aligning?	Third party tools	Parallelised?	Implementation
RetroSeq (Keane et al. 2013)	Transposable elements	Discordant reads, then split reads	No	No, but must be in BAM	SAMtools (v0.9), bcftools, exonerate, BEDtools	No	Perl
Tangram (Wu et al. 2014)	Transposable elements	Split reads and discordant reads simultaneously	Yes^a^	Yes, MOSAIK	MOSAIK (2.0), zlib, pthread lib	Yes	C, C ++
VirusSeq (Chen et al. 2013)	Viruses	Unmapped reads for general detection; Discordant and split-reads for integration site detection	No	Yes, MOSAIK	MOSAIK (0.9.0891)	Yes	Perl, C, C ++
MELT (Sudmant et al. 2015)	Transposable elements	Discordant reads, then split reads	Detects deletions	No, but must be in BAM	Bowtie2	No	Java
VirusFusionSeq (VFS) (Li et al. 2013)	Viruses	Unmapped reads for general detection; Discordant and split-reads for integration site detection	Yes^a^	Yes	BWA, SAMtools, BLAST,CAP3, SSAKE	Partially (BWA portion)	Pipeline (Perl)
Tlex2 (Fiston-Lavier et al. 2015)	Transposable elements	Looks at host and annotated TE flanks. Searches for split reads	Only detects in reference	No	MAQ, SHRIMP, BLAT, RepeatMasker, Phrap	Partially (MAQ)	Pipeline (Perl)
STEAK	Transposable elements and viruses	Split-reads, retrieves mate for PE data	Yes	No	Aligner of choice, BEDtools	Yes	C, C ++

a Can detect in reference but was not designed to mark presence-absence of reference insertions.

## 2. Results

### *2.1* *STEAK* algorithmic overview

**Figure 1.**
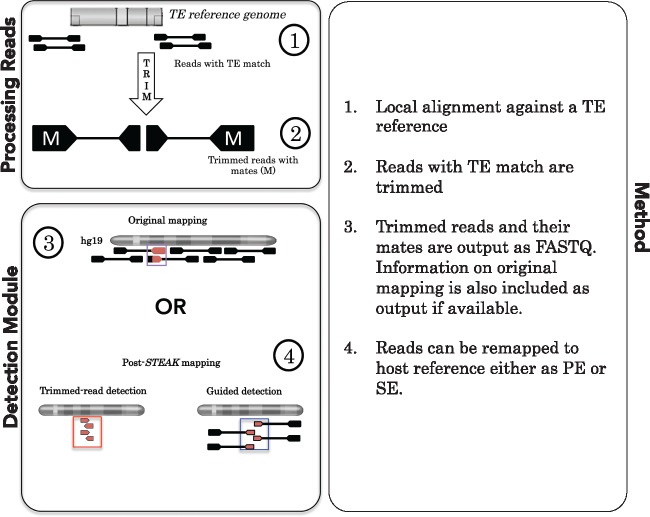
Workflow of STEAK. *Processing data:* All reads are locally aligned using the Smith Waterman algorithm and allowing mismatches when mapping reads against a TE reference (5′- and 3′-ends and respective reverse complements). Reads that match with the TE are trimmed of the matching portion. Information on the trimmed reads and their mates, such as the original mapping positions, MAPQ, and sequence qualities, are kept in *STEAK* outputs. *Detection Module:* Trimmed reads can be remapped to the human reference either as single-end (trimmed read detection) or paired-end reads (guided detection).

Our program firstly detects reads from a HTS library that contains fragments of a minimum similarity to a given short sequence (which can be the edge of a TE or a virus, hereafter called chimeric reads) and subsequently removes this fragment to produce a library with reads that only contain host reference flank (hereafter called trimmed reads). If the HTS library contains paired-end mates, then the respective paired-end mates of the trimmed reads are retrieved (Fig. 1). Novel integrations are often sufficiently random to produce unique chimeric reads however, in scenarios where the chimeric reads are not unique, these respective paired-end mates can be used to further support a novel integration. Users can then identify the location of an integration either from previous mapping information or by mapping trimmed reads to the host reference. Trimmed reads can be mapped either as single-end reads or with their respective mates. The latter, where trimmed reads and their mates are mapped as pairs, is referred to as guided detection and can be used to increase the possibility for uniquely mapping the trimmed read. For example, if the trimmed read comes from a highly repetitive region the mapping of the trimmed read alone will not be unique, but the combination of the mate and the trimmed read could provide a unique mapping solution. The outputs can then be used for custom downstream analyses most importantly for the reconstruction of preintegration sites, which is an excellent bioinformatics alternative to wet-lab verification of novel integrations. *In silico* verification is of paramount importance as full-genomes are becoming increasingly available while their original DNA samples are either not accessible or too valuable to be used for multiple PCR-guided verifications.

### 2.2 Input data

*STEAK* requires HTS data and a reference sequence that is expected to contain the edges of the mobile element (e.g. beginning and end of an LTR in the case of HERVs). Input HTS data can be either mapped (SAM) (Li et al. 2009) or raw (FASTQ). Compressed SAM, also known as BAM files, can be input for *STEAK* using tools such as SAMtools or biobambam2 (Tischler and Leonard 2014). TE and retroviral reference files must be in FASTA format*. STEAK* performs on both single-end (SE) and paired-end (PE) libraries and on a variety of HTS sequencing strategies. All input PE data should be collated by name for respective mate retrieval.

### 2.3 Chimeric read detection and trimming

The chimeric read detection and trimming phase of *STEAK* is a multistep process to identify and process reads, which contain bits of the retroviral or TE reference. *STEAK* takes the edges of the TE or retrovirus reference and creates reverse complements, producing four baits of a given length. With these baits, it looks for similarities by first aligning each read against the TE/virus bait with the Smith–Waterman algorithm (Zhao et al. 2013). Parameters can be modified to alter the length of minimum bait match sought for in a read as well as the per cent identity between reference and chimeric read. For example, current default parameters are of a 15-bp length bait and a 95% identity however, to search for HK2 integrations of five million years or younger, we used we used a bait length of 20 bp and a per cent identity of 90 to allow the detection of integrations which have mutated over time.

If the HTS data provided is already mapped (BAM/SAM), filtering based on the percentage of matches within a CIGAR value is also an available option for detecting chimeric reads. Filtering by CIGAR value is useful for non-reference TE discovery because reads pertaining to novel integrations are expected to have less than perfect mapping to the host. With this filtering, *STEAK* searches for any reads matching less than perfect with the original host reference (e.g. 94M7S for a 101-bp read or 6S280M15S for a 301-bp read), which can speed up processing significantly.

To annotate reference TE integrations, no CIGAR filtering is needed because reference integrations are expected to have reads mapping with perfect matches. In this case, *STEAK* will automatically search for chimeric reads that support integrations both present and not present in the host reference.

### 2.4 TE and retrovirus detection module

While there are a number of transposable element discovery pipelines (Ewing 2015), *STEAK* is capable of finding reads supporting both reference and non-reference TE and retroviral integrations. *STEAK* is a parallelised software that can function as a standalone or coupled with other tools for custom downstream analyses.

If the initially provided HTS data is already mapped, *STEAK* is able to retrieve information of the original mapping for the chimeric reads it detects. This on its own can often provide supporting reads for integrations, which is already seen with systems like *MELT* (Sudmant et al. 2015) and *RetroSeq* (Keane et al. 2013) (Table 1). But, in addition to this, the *STEAK* algorithm outputs trimmed reads which can be exploited to detect both reference and non-reference integrations. The advantage of trimming chimeric reads is that it allows for re-mapping of the host flanks to the original host genome forming clusters. These clusters of host-trimmed reads can then indicate the site of an integration. Furthermore, by providing the mates of trimmed reads, it is possible to perform guided detection (Fig. 1) where the mate can further support the proper mapping of a trimmed read.

The outputs from *STEAK*’s operations include (1) host trimmed reads and respective mates in FASTQ format, (2) the respective TE or retrovirus match in FASTQ format, and (3) a tab-delimited file providing information on the chimeric reads detected such as length of match, per cent identity, and previous mapping coordinates.

To detect these integration sites, we aligned host trimmed reads using *Novoalign* (Hercus 2009). We chose Novoalign like Marchi et al. (2014) because it is an accurate aligner particularly when dealing with single-end reads as it uses NeedlemanWunsch algorithm with affine gap penalties when scoring mapping locations. Single-end mapping was performed with default parameters. Paired-end alignment parameters were specified as end-to-end mapping with no soft clipping. Remappings were done using the host reference genomes originally used.

Our choice of downstream analyses consisted of using a combination of BEDtools (Quinlan and Hall 2010) and command line utilities, such as AWK and grep. To detect integration sites within the host reference, we provided a TE annotation file from *RepeatMasker* where the coordinates and the names of the known TE integrations are supplied. For known non-reference integrations, a TE annotation file compiled from the known literature is most appropriate. To mark presence or absence of known integrations, we compiled a list (Supplementary Table S3) made from *RepeatMasker* annotations of HK2, Subramanian et al. (2011), Marchi et al. (2014), Lee et al. (2012), and Wildschutte et al. (2016). For other transposable elements, users can provide a BED file of known reference TE insertions or use the *RepeatMasker* annotations (Tarailo-Graovac and Chen 2009).

Whereas for novel integrations, a list of both known reference and non-reference integrations should be used to ascertain that it is in fact a novel integration. Additionally, we filtered out other transposable elements that shared sequence similarity with HK2 LTR, such as Sine/VNTR/Alu (SVAs). We excluded clusters matching the non-HK2 LTR part of an SVA or which were in close proximity, within 1,000 bp, of a known HK2 or SVA locus (regions annotated in *RepeatMasker*). We considered a novel integration discovery when five reads or more were found clustered within a range of 10 kb. For non-endogenised elements, no annotation file is needed for integration discovery.

Detected candidate loci are output in two BED files: one for novel integrations and another for detection of known integrations. The BED files give coordinates of the region, the number of trimmed reads found for that locus, and if within another repetitive element. For target site duplication (TSD) identification, we used *BreakAlign* (Marchi et al. 2014) and *Geneious* (Kearse et al. 2012) (Supplementary Fig. S1) was used for preintegration reconstruction which is an *in silico* alternative of verifying an integration without wet-lab validation.

### 2.5 Software specifications and parallelisation

**Figure 2.**
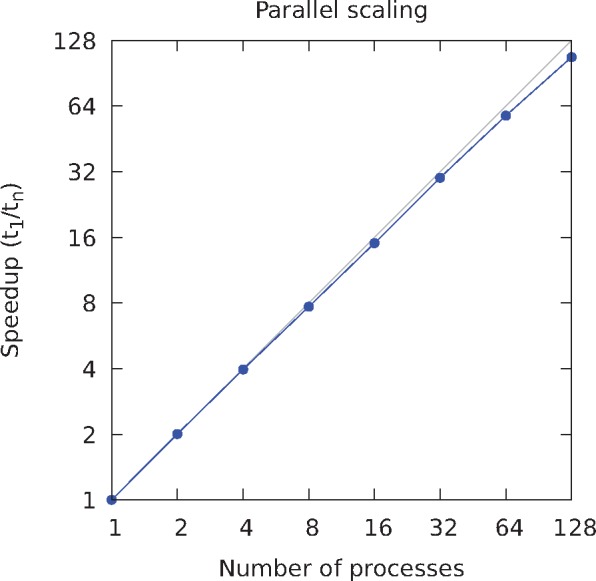
Parallelisation of *STEAK* processing. A 50× coverage simulation of chromosome 1 was processed using our MPI-based software. The speedup as a function of the number of cores shows that the program scales well because few concurrency issues affect it.

**Figure 3.**
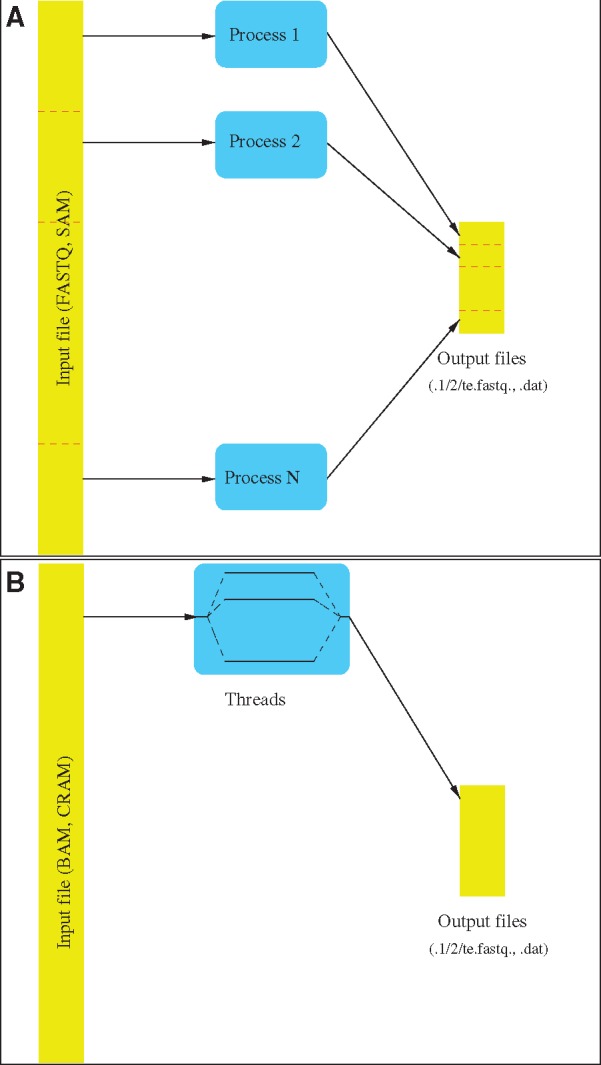
Processing in *STEAK.* (**A**) The software runs as several processes that read different parts of the input SAM or FASTQ file. (**B**) In the case of a BAM/CRAM file, because the data is read from a single point, a unique process is executed but it spawns several threads, one that accumulates the data in a buffer and the others that process them. TEs are specified in a FASTA file. The trimmed sequence is written in the first FASTQ file. The original sequence of the other read (or the trimmed sequence if there is a match as well) is placed in the second FASTQ file.

*STEAK* has been programmed in C ++ and been designed to be massively parallel, meaning that it is not limited to a single computer. This enables *STEAK* to run efficiently on large high-performance computing clusters and to process quickly high-coverage genomes (Fig. 2). The input file is split in different parts that are each handled by a different process, using MPI (Fig. 3A). The software is afflicted by very few concurrency issues and, as a consequence, should scale well until the limits arising from the file system are reached. Unfortunately, this parallelisation strategy is not possible when we endeavour to process a BAM file because, in that case, the data are accessed sequentially, through a single point. This is because we are processing the output of the decompression fed through a pipe, i.e. the redirection of a standard output, and therefore, the whole file is not accessible to be read from several distinct locations. To cater to that possibility, we have resorted to multithreading (Fig. 3B). A thread reads the input file and accumulates the reads in a circular buffer while several other threads process those reads in parallel. This leads to a noticeable performance improvement on fast file systems.

### 2.6 Overview of features and comparison to other TE and virus software

Our program works on re-sequencing projects and therefore additional downstream analysis requires good quality, well-annotated host reference genomes in addition to reference TE or retrovirus sequences (i.e. the TE sequence expected to be at an integration site). It is dependent on existing mapping software; for example, in order to identify potential retroviral integrations in a human genome, we would need to provide the human reference genome as well as the edges of the suspected retroviral LTRs. While the HTS data need not be mapped initially, another mapping program is required to detect integration sites (see Availability and Requirements). We pinpoint four important features of *STEAK* that to the best of our knowledge are combined in a package for the first time: firstly, it allows for detection of the TE (or viral) integration even if there has been deterioration of the sequence through time; secondly, it locates the absence of TE sequences that exist in the provided reference genome; thirdly, it facilitates a vast number of downstream analyses (i.e. the reconstruction of novel integrations); and fourthly, it successfully works on a variety of sequencing projects including target enrichment. Several other features of *STEAK* in comparison with other existing software are provided in Table 1. While we are aware of a number of other software that exist for transposable element (Ewing 2015) and virus detection, we have chosen those which have comparable features, popular usage, and are currently competitive (see Supplementary Note S1 and Supplementary Table S1).

### 2.7 Evaluation of STEAK

For our benchmarking, we firstly tested the ability of STEAK to identify HK2 integrations in human full-genome re-sequencing HTS projects. We set bait length parameters to be 20 bp of the beginning and end of the LTR (both strands) for mining out chimeric reads (K113, Accession Number: NC_022518.1). A match between this 20-bp bait and each read was searched by means of the Smith–Waterman algorithm (Zhao et al. 2013) which allows for indels and substitutions between the TE/virus reference and the read. This local alignment filtering only permitted reads through a certain threshold, allowing a limited number of mutations between the LTR reference and a read when searching for a 20-bp match (e.g. 90% similarity = up to two mismatches). Reads that passed filtering were trimmed of the LTR matching sequence (Fig. 1). We only kept trimmed reads with a minimum length of 20 bp. Trimmed read length and TE match lengths are adjustable parameters in *STEAK.*

**Figure 4.**
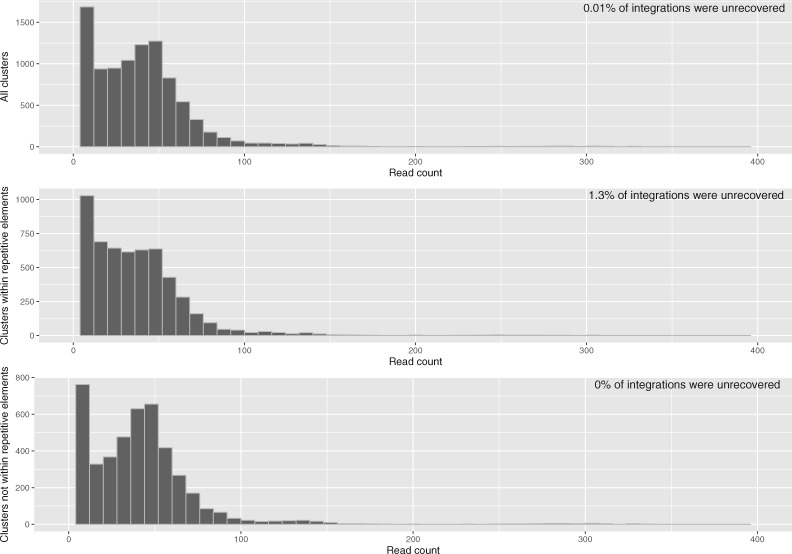
Distributions for cluster read depth of known HK2 integrations. *Top:* Cluster read depth for all known integrations. *Middle:* Cluster read depth for known integrations within repetitive elements. *Bottom:* Cluster read depth for known integrations that are not within repetitive elements.

We initially tested *STEAK* on seventy WGS from The Cancer Genome Atlas (Supplementary Table S2) to find presence and absence of already characterised HK2 integrations. We compiled a list of HK2 proviruses and solo LTRs that are five million years old or younger and can therefore be polymorphic in the human population (Supplementary Table S3). From this list of 183 HK2 integrations, we used the 133 known fixed HK2 integrations to observe the depth of reads for present HK2 integrations amongst the 70 samples. Based on the cluster of trimmed reads found for these known integrations, we determined an appropriate threshold to consider a potential novel integration (Fig. 4).

Our screening showed that some known fixed HK2 integrations were more difficult to recover than others. For example, integrations that are within other repetitive elements were less likely to be picked up by our program, 1.3% of integrations within repetitive elements were unrecovered (Fig. 4). Integrations with flanking repetitive regions may require lowering the tolerance below the 90% identity threshold we used or multi-location mapping can potentially resolve ambiguous trimmed read mappings. To account for these difficult regions and to increase our sensitivity, we only considered clusters with a minimum of five reads as potential novel candidates. However, for already known and characterised HK2 integrations in the literature (both reference and non-reference), we accepted single reads as evidence of presence. In spite of the difficulty that TEs within repetitive regions may pose, only one of the seventy-seven HK2 integrations within a repetitive element was unrecovered by *STEAK*, all the other 132 integrations were recovered. Other regions that pose difficulties in recovering integrations include pseudogenes or genes with multiple copies in the genome (Supplementary Note S2 and Supplementary Fig. S1).

### 2.8 Performance evaluation on simulated data

We evaluated *STEAK*’s ability to detect both TEs and retroviruses with a series of computational experiments. The first sets of experiments were done using *Tangram*’s original Alu simulations into chromosome 20 and another custom simulation where HK2 integrations were inserted into chromosome 20 for benchmarking purposes (Supplementary Table S4). This provided insight into the positive predictive value (PPV), or the probability that a suggested integration was true, for *STEAK*, *Tangram*, and *RetroSeq*. When using only single-end trimmed read mapping, *STEAK* performed less sensitively but with higher PPV than the two other software (Supplementary Fig. S2A and B).

**Figure 5.**
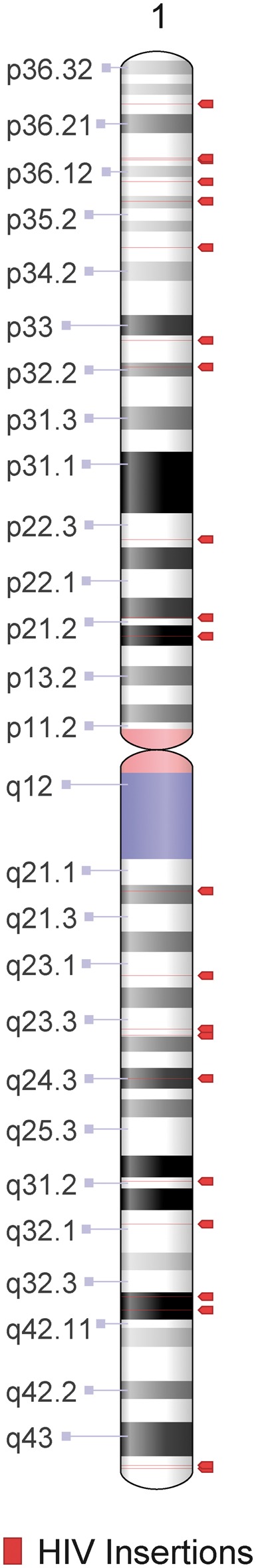
Distribution of twenty simulated HIV integrations within human reference (hg19) in chromosome 1. Respective genomic coordinates can be found in Table 2.

For further evaluation of *STEAK*’s ability to detect integrations, in particular retroviruses, we created a simulation based on hg19 chromosome 1 with 20 full-length HIV integrations (Fig. 5) and with simulated reads that match realistic Illumina sequencing errors (Methods). With this simulation, we evaluated the different forms of detection that STEAK has to offer: using original alignment information, using trimmed read detection, and using guided detection. Guided detection performs the most sensitively in its ability to recover all simulated integrations with the most amount of supporting reads per integration within a 100-bp window from the original simulated insertion site (Table 2). These results suggest that while trimmed reads can provide specific integration detection, if mapped alone they lose out on the sensitivity that being paired offers in mapping. Similarly, *VFS* was also able to detect all simulated integrations by making use of both chimeric reads and discordant pairs although with a substantially slower pipeline (Supplementary Note**S**1).

**Table 2.** Twenty simulated HIV integrations into human chromosome 1.

Human reference (hg19)		Original mapping	Post-trimming	
Chromosome	Position	Supporting reads	Guided detection	Trimmed read detection
1	4179520	35	100	64
1	10331435	34	113	70
1	16830086	30	96	57
1	18869777	31	112	67
1	20389049	36	76	45
1	54327146	34	93	63
1	57730318	34	101	70
1	99180019	24	92	53
1	116586277	31	85	54
1	144993094	16	18^a^	0^a^
1	149062299	36	99	63
1	165127302	43	109	71
1	170764099	15	69	44
1	188462855	43	121	85
1	191791184	25	80	53
1	197518001	34	88	55
1	213559631	26	90	52
1	219498833	37	97	63
1	223662699	38	95	61
1	231971371	26	78	44

a Where trimmed reads alone could not detect the integration.

### 2.9 Performance on whole genome and target enrichment data

We screened samples of whole genome sequence datasets provided by The Cancer Genomes Atlas Project (TCGA), the 1000 Genomes Project, and a target-enrichment project. The CEU pedigree, four patient genomes, and the target enrichment sample are listed in Table 3. These samples were chosen for their high coverage and representativeness of different projects that are publically available (e.g. 1000 Genomes Project) as well as clinical (e.g. TCGA). The CEU pedigree was chosen because it is one of the best-sequenced pedigrees, which is also publically available making it ideal for benchmarking purposes.

**Table 3.** The sequencing samples analysed in benchmarking.

Sample	Dataset	Coverage
TCGA-A6-2681-10A-01D-2188-10 (COAD)	TCGA: Colon adenocarcinoma	50×
TCGA-HC-7233-10A-01D-2115-08 (PRAD)	TCGA: Prostate adenocarcinoma	50×
TCGA-NJ-A4YQ-10A-01D-A46J-10 (LUAD)	TCGA: Lung adenocarcinoma	50×
TCGA-BW-A5NQ-10A-01D-A27I-10 (LIHC)	TCGA: Liver hepatocellular carcinoma	45×
NA12878	1K Genomes: CEU pedigree (Offspring)	50×
NA12891	1K Genomes: CEU pedigree (Father)	50×
NA12892	1K Genomes: CEU pedigree (Mother)	50×
HK2_Enrich01	NA	500×
HK2_Enrich02	NA	500×

All TCGA samples were DNA derived from peripheral blood and were sequenced with Illumina platform for whole genome sequencing. CEU pedigree samples derived from immortalised cell lines maintained by 1000 Genomes Project and were sequenced with Illumina platform for whole genome sequencing. Target enrichment samples were germline derived and sequenced as described in the methods section.

With *STEAK*, we are able to mark the presence and absence of both reference and non-reference integrations. For comparison purposes, we benchmarked *RetroSeq* and *MELT* using non-reference integrations. For benchmarking presence and absence of reference integrations, we compared our results to *MELT*.

**Figure 6.**
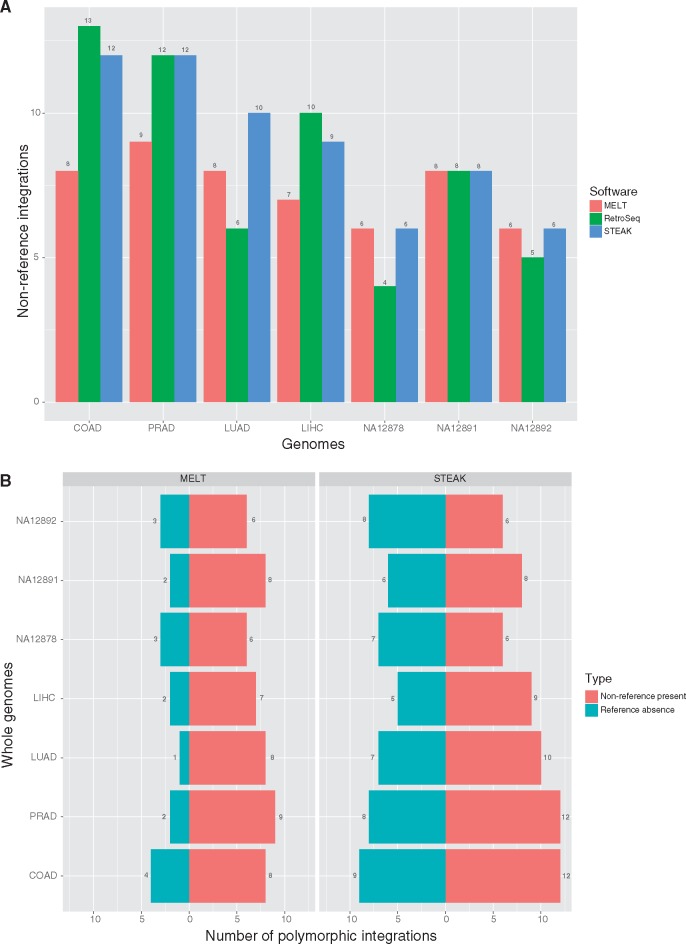
Comparative performance of HK2 detection in whole genome sequencing. (**A**) Bar graph displays known non-reference integrations detected in WGS projects by each respective system. (**B**) Pyramid plot depicts number of polymorphic integrations detected per genome—number of present non-reference and number of absent reference integrations.

We observed that *RetroSeq* and *STEAK* often exceeds the sensitivity of *MELT* for non-reference integrations (Fig. 6A) and that *STEAK* performs more sensitively than *MELT* in detecting known polymorphic HK2 integrations regardless of whether they are reference or non-reference (Fig. 6B). We also intended to compare our reference integration results to *tlex2* results but, *tlex2* is unable to handle high-coverage genomes (Supplementary Note S1).

**Figure 7.**
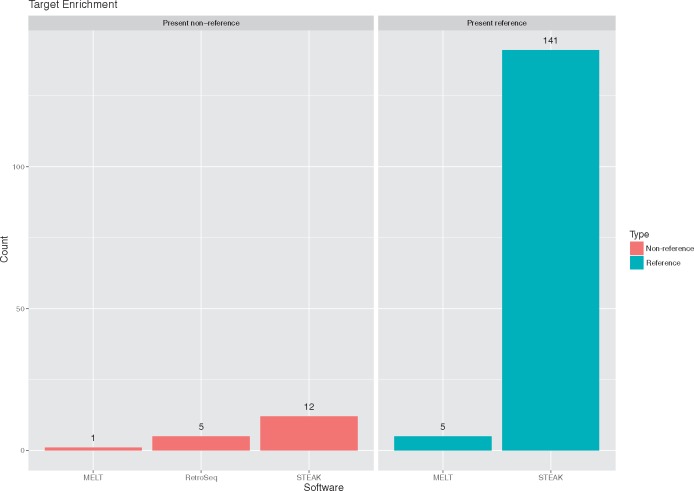
Detection performance in target enrichment data. The left facet depicts presence of non-reference integrations detected by each respective system. The right facet depicts the marking of presence of reference integrations. *MELT* results detect 5 reference presences and 137 absences.

Crucially, *STEAK* clearly demonstrates its ability to handle long fragment-sized libraries, such as target-enrichment data, and significantly outperforms *MELT* and *RetroSeq* in detecting polymorphic integrations both in and not in the reference genome (Fig. 7). In the case of MELT’s deletion genotyping module, it marks 137 reference integrations, of which a great majority tend to be fixed within the population, as absent in the target enrichment data.

## 3. Discussion

The driving motive behind developing *STEAK* was to detect polymorphic endogenous retrovirus integrations in HTS data with high coverage. The overarching difference of *STEAK*, compared to previous algorithms that we have published (Marchi et al. 2014), was that it allowed for the potential evolution of the transposable element or virus by tolerating for a controllable number of mutations to be present in the reference sequence. This latter part is computationally intensive, thus we accelerated the process by massively parallelising our software.

*STEAK* has been designed to include functionality features that are largely missing from other similar available software. *RetroSeq, MELT*, and *TEA*, rely on paired-end data and work on discordant or unmapped mates (Li et al. 2009). Those algorithms cannot work with single-end reads, longer-read libraries where pairs partially overlap, and do not provide outputs for users to continue their own downstream analysis. While *MELT*, *RetroSeq*, and *TEA* provide the coordinates for TSD breakpoint intervals, they do not provide outputs for reconstructing pre-integration sites or to further characterise the novel integrations found, all of which can be particularly useful when processing whole genome sequences without access to original DNA samples. This is highlighted in clinical datasets, like TCGA, which often include data from individuals that have been sequenced with >30× coverage but have restricted access to samples. In such cases, the ability to recover supporting chimeric reads for TEs or retroviral integrations in high-coverage genomes is valuable. One example would be using WGS to observe the changes in the TE profile of an individual, such as an abundance of a TE in tumour tissue when compared to the germline (Criscione et al. 2014). On the other hand, screening deep sequencing target enrichment experiments can be equally as crucial for clinical research: for example, target enrichment is a cheaper alternative to WGS, particularly if there is a need to sequence multiple samples to observe a specific active retrotransposon or retrovirus. In this regard, *STEAK* was the only software capable of sensitively retrieving integrations from both WGS and target enrichment sequencing projects (Figs 6 and 7).

Our program will efficiently process both mapped (SAM/BAM) and unmapped (FASTQ) HTS data, paired and single-end sequencing projects, and whole genome sequencing as well as target enrichment strategies. *STEAK* is also the only program that has an integrated approach for users to detect reads that support TE and retroviral integrations: using original mapping information, trimmed read mapping and guided detection. Moreover, it is the only software flexible enough to provide users with outputs to move forward with custom downstream analysis.

How does *STEAK* compare with other similar software with respect to performance? We compared *STEAK* with *MELT*, *RetroSeq*, and *Tangram* on paired-end datasets (which in principle can be handled by all of them). *RetroSeq* and *Tangram* fared well in sensitively detecting TEs within low-coverage genomes (Rishishwar et al. 2016), but we observed an increase of false-positives when used for discovery in deep-coverage WGS (Supplementary Fig. S2A and B). Such programs can be used with low-coverage WGS but the verification of the breakpoint would need to go through a wet-lab approach. *STEAK* performs better than other TE discovery software when recovering specific integrations with high confidence, which is the case with individual deep-coverage genomes. It is a program that can both specifically and sensitively mark presence or absence of reference and non-reference TEs with its adjustable parameters. We observed that certain HK2 proviruses were more difficult to recover by *STEAK* when integrated within other mobile elements. When such proviruses within repetitive elements are already described (i.e. no verification is required) we can increase the sensitivity of our downstream analyses by accepting the presence of the provirus even with a single chimeric read spanning the known junction. As demonstrated in the simulation (Table 2), the guided detection that *STEAK* facilitates allows rescuing of integrations where host trimmed reads do not map uniquely. However, when these proviruses are not catalogued an approach like *RetroSeq* would be more likely to recover them as potential candidates (Fig. 6A); although these candidate integrations are likely to need wet-lab verification, as the PPV of *RetroSeq* remains low (Supplementary Fig. S2B).

*STEAK* stands out in its ability to identify more integrations within other forms of sequencing strategies, such as target-enrichment data, in comparison to already existing systems (Fig. 7). Numerous sequencing platforms are making efforts to output longer reads which will inevitably provide longer reads flanking the integration junctions. *STEAK* was designed with this in mind and already proves to handle such sequencing strategies without any difficulties.

The limitations of *STEAK* in comparison to other software, like *MELT* and *RetroSeq*, lie in that it does not mark zygosity of an integration and that it does not calculate the exact breakpoint interval. In our analyses of non-reference HK2 integrations, *STEAK* provides flexible outputs to easily recur to software such as *BreakAlign* (Marchi et al. 2014) and *Geneious* (Kearse et al. 2012) for TSD detection and pre-integration site reconstruction (Supplementary Fig. S1).

We show that *STEAK* performs as well as or better than competitive systems available when detecting non-reference integrations (Fig. 6B). As an algorithm and software, it is remarkable in its flexibility to accept a variety of HTS data and process high-coverage genomes in a parallelised manner where other systems struggle or even fail (Supplementary Note S1). The purpose of *STEAK* is to fill a gap that exists in regards to detecting mobile elements (either virus or transposable elements) while also providing adjustability in its detection strategies and assisting users in their custom downstream analyses.

## 4. Conclusions

*STEAK* is a tool that detects integrations of any sort in HTS datasets with higher sensitivity and specificity than existing software, and can be applied to a broad range of research interests and clinical uses such as population genetic studies and detecting polymorphic integrations.

## 5. Methods

### 5.1 General benchmarking parameters

We ran *RetroSeq* and *Tangram* under the instructions provided on their respective Github sites. For *VirusSeq*, we benchmarked using the instructions from the User Manual (Chen et al. 2013). We ran *STEAK* using the parameters provided in the supplementary data. This along with the command lines for the software we benchmarked against can be found in Supplementary Note S4.

### 5.2 WGS from the Cancer Genome Atlas

We analysed four normal blood derived samples (‘germlines’) from patients that were available at an average range of 45× to 50× coverage. These paired-end WGS (2 × 100 bp) were all sequenced using Illumina Genome Analyzer platform technology (Table 3). All whole genomes downloaded from TCGA database were pre-aligned to the human reference, version hg19.

### 5.3 WGS of CEU (Utah Residents with Northern and Western European Ancestry) pedigree from the 1000 Genomes Project

We analysed the Illumina platinum genomes for the NA12878/NA12891/NA12892 pedigree. These samples were WGS sequenced at a 50× coverage with both paired-end (2 × 100 bp) and single-end libraries with Illumina HiSeq 2000 system. All whole genomes were downloaded from 1000 Genomes Project data portal.

### 5.4 Simulated datasets and benchmarking

For the TE-simulated dataset, we tested on a simulated human chromosome 20 of 5× coverage and a read length of 76 bp created by Wu et al. (2014) in the release of *Tangram*. We compared *VirusSeq* (Chen et al. 2013), *RetroSeq* (Keane et al. 2013), *Tangram* (Wu et al. 2014), and *STEAK* on the detection sensitivity of AluY non-reference insertions in this simulated chromosome 20 data. The second simulated genome we benchmarked was one we produced from manually inserting ten HK2 LTRs into chromosome 20 across the genome (Supplementary Table S4). Using the MASON read simulator, we created an Illumina paired-end WGS dataset with 50× coverage and reads of 101-bp length. We mapped these reads to the hg19 reference using BWA. The resulting genome was benchmarked with *RetroSeq, VirusSeq*, and *STEAK*. *Tangram* runs on MOSAIK aligned genomes and did not accept any of our BAMs produced by other aligners in spite of a program that was released to add the necessary ZA tags (tangram_bam). Furthermore, *Tangram* is currently unmaintained and unsupported.

*RetroSeq, Tangram, VirusSeq*, and *STEAK* were all run with matching parameters to compare sensitivity and specificity on a simulated chromosome 20 with artificial HK2 insertions.

For the HIV-simulated dataset, we based it on the human genome reference (hg19) chromosome 1. Twenty full-length HIV integrations were randomly introduced into chromosome 1 using VirusFusionSeq viral insertion simulator (Li et al. 2013). The full-length HIV insertion was taken from the HIV1/LAV reference (Accession number: K03455.1). We created simulated Illumina paired-end reads with the ART next generation sequencing read simulator (Huang et al. 2012) with default error model and indicating 50× coverage and 100-bp reads. The reads were then aligned to hg19 using BWA MEM.

### 5.5 Target-enrichment dataset

We also tested *STEAK* on a sample that was prepared through targeted enrichment of the ends of HK2 LTR. Briefly, DNA was extracted from control Novagen™ human genomic DNA. Genomic regions of interest were selected using a biotin-streptavidine-based bead capture with DNA bait probes. In this case, target-specific baits used came from the beginning and end of HK2 LTR (K113)— ∼360 bp from each end. Five overlapping probes were used for each end; each probe was 120 bp in length. Single-stranded oligonucleotides with a common linker flanked by target-specific sequences anneal to the sequences of interest and capture them (Gnirke et al. 2009; Mamanova et al. 2010). After target enrichment hybridisation, the sample was sequenced using the Illumina MiSeq platform producing PE 300-bp paired-end reads.

### 5.6 Availability and requirements

*STEAK* relies on boost-libraries, OpenMP, gcc, python, and BEDtools. SAMtools or biobambam2 can be used to decompress BAM files. It has purposely been designed to use as little dependencies as possible for negligible installation hassle. Trimmed reads can be processed with an aligner of choice—we recommend a sensitive mapper such as *Novoalign * (Hercus 2009), BWA MEM (Li and Durbin 2010) or Stampy (Lunter and Goodson 2011). 

## Supplementary Material

Supplementary Data

Supplementary Table 5

## Data Availability

*STEAK* is publically available through Github (http://github.com/applevir/STEAK) with an accompanying test dataset and instruction manual. Details to create the simulated genomes of 50× coverage can be found in the Methods section. The 5× coverage genome can be found on *Tangram*’s GitHub repository. TCGA WGS data were retrieved through the Genome Data Portal (https://gdc-portal.nci.nih.gov/legacy-archive/search/f) after applying for access to controlled-access data through dbGAP authorisation. The CEU pedigree WGS data were retrieved through the 1000 Genomes beta Data Portal (http://www.internationalgenome.org/data-portal/sample) under Illumina Platinum pedigree data collection. The target enrichment library sequences data from this study have been submitted to the ENA short read archive under the accession number PRJEB21477; Sample accession numbers are ERS1796985-6 inclusive.
