# Supplementary material for: STEAK: A specific tool for transposable elements and retrovirus detection in high-throughput sequencing data
Source: Virus Evol. 2017 Aug 21;3(2):vex023. doi: 10.1093/ve/vex023 (PMC5597868; doi:10.1093/ve/vex023)
Supplement: Supplementary Data [file vex023_supp.docx]

**Title**: STEAK: a specific tool for transposable elements and retrovirus detection in high-throughput sequencing data.

**Authors**

Cindy G. Santander^1,*^, Philippe Gambron^2^, Emanuele Marchi^3^, Timokratis Karamitros^1^, Aris Katzourakis^1,^, and Gkikas Magiorkinis^1,4*^

**Affiliations**

1. Department of Zoology, University of Oxford, Oxfordshire, United Kingdom
2. Science and Technology Facilities Council, Rutherford Appleton Laboratory, Harwell Science and Innovation Campus, Didcot, Oxfordshire, United Kingdom
3. Nuffield Department of Medicine, University of Oxford, Oxfordshire, United Kingdom
4. Department of Hygiene, Epidemiology and Medical Statistics, Medical School, National and Kapodistrian University of Athens, Greece

^*^ Correspondence to (G.M.)  [gkikasmag@gmail.com](mailto:%20gmagi@med.uoa.gr) and (C.S.) [cindy.santander@zoo.ox.ac.uk](mailto:aris.katzourakis@zoo.ox.ac.uk)

**This PDF file includes the following supplementary material:**

- Supplementary Notes 1, 2, 3, and 4
- Supplementary Tables 1, 2, 3 and 4
- Supplementary Figures 1 and 2

**Note 1**

**Benchmarking software for comparative evaluation**

STEAK was written with the intention of looking for LTR transposons such as HERVs. The algorithm designed was meant to detect and visualise TE insertions as well as retroviral integrations. Our decision to use *RetroSeq* (Keane et al. 2013)*, Tangram* (Wu et al. 2014) and *VirusSeq* (Chen et al. 2013) rose from how widespread their usage is (Table S3), what features they represent, and how appropriate they were in detecting HERV-K. *RetroSeq* is written in Perl and makes use of paired-end reads to determine novel TE integrations.

*Tangram* is a multithreaded program written in C++ and was designed to look for TE in both PE and SE libraries. Unfortunately it is no longer maintained and has difficulties handling BAM files not aligned with a specific version of *MOSAIK*.

*VirusSeq* is a pipeline that makes use of *MOSAIK* (Lee et al. 2014) (C++) and scripts written in Perl to determine virus presence and detect integration sites. However it was unable to recover HERV-K (HML-2) integrations (Note 3).

Whilst benchmarking for presence/absence with *t-lex2* (Fiston-Lavier et al. 2015) we discovered that it is unable to handle high-coverage genomes. We ran on ARC facilities tlex2 pipeline on NA12878 using 256 GB of memory, 16 processes after replacing blat with pblat in the *tlex2* pipeline with several runs that exceeded and timed-out after 120 hours. Worryingly, *tlex2* does not produce errors or warnings when it runs out of memory and also produces differing batches of intermediate files depending on the memory availability suggesting that it was not meant to handle copious amounts of HTS data.

*ViralFusionSeq* (VFS) (Li et al. 2013) is a competitive virus detection pipeline with its implementation in Perl. On the simulation of chromosome 1 it detected all simulated HIV integrations created with their own simulator. However, VFS like *tlex2* is unable to handle high coverage HTS and is mainly meant for RNA-Seq data. Similarly VFS timed out on NA12878 and did not complete searching for HERV-K in 120 hours.

The literature for *t-lex2* and VFS suggest that these pipelines have high sensitivity and specificity but their implementation and file handling are best suited for low coverage genomes.

Other relevant software include *Mobster* (Thung et al. 2014) and *Jitterbug* (Henaff et al. 2015). *Mobster* and *Jitterbug* are algorithms already similar to *RetroSeq* in that their main mission is to sensitively detect *de novo* TE insertions. Other pipelines like *TEA* (Lee et al. 2012)*, TraFiC* (Tubio et al. 2014)*,* and *TranspoSeq* (Helman et al. 2014) were not deemed appropriate because they tend to focus on tumour/normal pair which we do not analyse in our study.

Supplementary Table 1. Relevant software considered for comparative benchmarking.

| **Software** | **Year Published^a^** | **Year Released^b^** | **Number of Citations^*^** |
| --- | --- | --- | --- |
| RetroSeq | 2013 | 2013 | 41 |
| VirusSeq | 2013 | 2013 | 38 |
| VFS | 2013 | 2013 | 24 |
| Tangram | 2014 | 2014 | 12 |
| Mobster | 2014 | 2013**^c^** | 9 |
| T-lex2 | 2015 | 2012**^d^** | 9 |
| Jitterbug | 2015 | 2015**^e^** | 3 |
| MELT | 2015 | 2016**^f^** | Not available |

^a^ Year in which corresponding manuscript was published

^b^ Year that software was initially released and made available to public use

^c^ Retrieved from documentation on Sourceforge (2014).

^d^ Retrieved from documentation on T-lex web page (2011).

^e^ Retrieved from documentation on GitHub (2015).

^f^ Retrieved from documentation on MELT web page (2016).

^*^Citation record of corresponding manuscripts as provided by “Citation Network” in *Web of Science Core Collection.* (Reuters)

**Supplementary Table 2. The Cancer Genome Atlas 70 samples analysed in this study**

| **Sample** | **Disease** | **Centre** | **Assembly** | **Coverage** |
| --- | --- | --- | --- | --- |
| TCGA-DK-A1AG-10A-01D-A13W-08 | Bladder Urothelial Carcinoma | Broad Institute | HG19 Broad Variant | 39× |
| TCGA-HW-7486-10A-01D-2024-08 | Brain Lower Grade Glioma | Broad Institute | HG19 Broad Variant | 45× |
| TCGA-A2-A0D2-10A-01D-A128-09 | Breast invasive carcinoma | Washington University School of Medicine | GRCh37-lite | 39× |
| TCGA-A2-A04T-10A-01D-A128-09 | Breast invasive carcinoma | Washington University School of Medicine | GRCh37-lite | 39× |
| TCGA-EK-A2RM-10A-01D-A18J-09 | Cervical squamous cell carcinoma & Endocervical adenocarcinoma | Washington University School of Medicine | GRCh37-lite | 39× |
| TCGA-06-0145-10A-01D-0507-08 | Glioblastoma multiforme | Broad Institute | HG19 Broad Variant | 50× |
| TCGA-DQ-5625-10A-01D-A32X-10 | Head and Neck squamous cell carcinoma | Baylor College of Medicine | GRCh37-lite | 50× |
| TCGA-KM-8441-10A-01D-2311-10 | Kidney Chromophobe | Baylor College of Medicine | GRCh37-lite | 50× |
| TCGA-DV-5566-10A-01D-2104-10 | Kidney renal clear cell carcinoma | Baylor College of Medicine | GRCh37-lite | 45× |
| TCGA-MH-A562-10A-01D-A26P-10 | Kidney renal papillary cell carcinoma | Baylor College of Medicine | GRCh37-lite | 50× |
| TCGA-FF-8062-10A-01D-2210-10 | Lymphoid Neoplasm Diffuse Large B-cell Lymphoma | Baylor College of Medicine | GRCh37-lite | 50× |
| TCGA-FF-8046-10A-01D-2210-10 | Lymphoid Neoplasm Diffuse Large B-cell Lymphoma | Baylor College of Medicine | GRCh37-lite | 50× |
| TCGA-13-0725-10B-01D-0446-08 | Ovarian serous cystadenocarcinoma | Broad Institute | HG19 Broad Variant | 50× |
| TCGA-13-0751-10A-01D-0446-08 | Ovarian serous cystadenocarcinoma | Broad Institute | HG19 Broad Variant | 50× |
| TCGA-HC-7233-10A-01D-2115-08 | Prostate adenocarcinoma | Broad Institute | HG19 Broad Variant | 39× |
| TCGA-G9-6336-10A-01D-1786-08 | Prostate adenocarcinoma | Broad Institute | HG19 Broad Variant | 39× |
| TCGA-EE-A2MI-10A-01D-A199-08 | Skin Cutaneous Melanoma | Broad Institute | HG19 Broad Variant | 39× |
| TCGA-CD-5802-10A-01D-1600-08 | Stomach adenocarcinoma | Broad Institute | HG19 Broad Variant | 45× |
| TCGA-E8-A416-10A-01D-A23K-08 | Thyroid carcinoma | Broad Institute | HG19 Broad Variant | 50× |
| TCGA-AP-A053-10A-01D-A013-09 | Uterine Corpus Endometrioid Carcinoma | Washington University School of Medicine | GRCh37-lite | 39× |
| TCGA-A7-A0CE-11A-21D-A12L-09 | Breast invasive carcinoma | Washington University School of Medicine | GRCh37-lite | 50× |
| TCGA-A1-A0SM-10A-02D-A099-09 | Breast invasive carcinoma | Washington University School of Medicine | GRCh37-lite | 50× |
| TCGA-CH-5750-10A-01D-1576-08 | Prostate adenocarcinoma | Broad Institute | HG19 Broad Variant | 50× |
| TCGA-CH-5789-10A-01D-1576-08 | Prostate adenocarcinoma | Broad Institute | HG19 Broad Variant | 50× |
| TCGA-D3-A3MO-10A-01D-A21A-08 | Skin Cutaneous Melanoma | Broad Institute | HG19 Broad Variant | 50× |
| TCGA-BH-A0E0-11A-13D-A128-09 | Breast invasive carcinoma | Washington University School of Medicine | GRCh37-lite | > 50× |
| TCGA-CH-5763-11A-01D-1576-08 | Prostate adenocarcinoma | Broad Institute | HG19 Broad Variant | > 50× |
| TCGA-EE-A2M5-10A-01D-A199-08 | Skin Cutaneous Melanoma | Broad Institute | HG19 Broad Variant | 50× |
| TCGA-CH-5788-10A-01D-1576-08 | Prostate adenocarcinoma | Broad Institute | HG19 Broad Variant | 50× |
| TCGA-A6-2681-10A-01D-2188-10 | Colon adenocarcinoma | Baylor College of Medicine | GRCh37-lite | > 50× |
| TCGA-D3-A1Q5-10A-01D-A198-08 | Skin Cutaneous Melanoma | Broad Institute | HG19 Broad Variant | 50× |
| TCGA-06-0125-10A-01D-1490-08 | Glioblastoma multiforme | Broad Institute | HG19 Broad Variant | > 50× |
| TCGA-A6-2683-11A-01D-1554-10 | Colon adenocarcinoma | Baylor College of Medicine | GRCh37-lite | > 50× |
| TCGA-FF-8041-10A-01D-2210-10 | Lymphoid Neoplasm Diffuse Large B-cell Lymphoma | Baylor College of Medicine | GRCh37-lite | > 50× |
| TCGA-FF-8042-10A-01D-2210-10 | Lymphoid Neoplasm Diffuse Large B-cell Lymphoma | Baylor College of Medicine | GRCh37-lite | > 50× |
| TCGA-FF-8043-10A-01D-2210-10 | Lymphoid Neoplasm Diffuse Large B-cell Lymphoma | Baylor College of Medicine | GRCh37-lite | > 50× |
| TCGA-FF-8061-10A-01D-2210-10 | Lymphoid Neoplasm Diffuse Large B-cell Lymphoma | Baylor College of Medicine | GRCh37-lite | > 50× |
| TCGA-X2-A95T-10A-01D-A37F-09 | Sarcoma | Washington University School of Medicine | GRCh37-lite | > 50× |
| TCGA-AP-A0LE-10A-01D-A10B-09 | Uterine Corpus Endometrioid Carcinoma | Washington University School of Medicine | GRCh37-lite | > 50× |
| TCGA-AP-A0L9-10A-01D-A066-09 | Uterine Corpus Endometrioid Carcinoma | Washington University School of Medicine | GRCh37-lite | > 50× |
| TCGA-AX-A1CI-11A-11D-A135-09 | Uterine Corpus Endometrioid Carcinoma | Washington University School of Medicine | GRCh37-lite | > 50× |
| TCGA-L5-A4OR-11A-11D-A27G-09 | Esophageal carcinoma | Washington University School of Medicine | GRCh37-lite | > 50× |
| TCGA-06-0185-10B-01D-0507-08 | Glioblastoma multiforme | Broad Institute | HG19 Broad Variant | > 50× |
| TCGA-06-0648-10A-01D-0507-08 | Glioblastoma multiforme | Broad Institute | HG19 Broad Variant | > 50× |
| TCGA-G3-A25T-10A-01D-A16V-10 | Liver hepatocellular carcinoma | Baylor College of Medicine | GRCh37-lite | > 50× |
| TCGA-IG-A3YB-10A-01D-A247-09 | Esophageal carcinoma | Washington University School of Medicine | GRCh37-lite | > 50× |
| TCGA-A6-3807-10A-01D-1459-10 | Colon adenocarcinoma | Baylor College of Medicine | GRCh37-lite | > 50× |
| TCGA-LN-A49M-10A-01D-A27G-09 | Esophageal carcinoma | Washington University School of Medicine | GRCh37-lite | > 50× |
| TCGA-06-0188-10B-01D-0373-08 | Glioblastoma multiforme | Broad Institute | HG19 Broad Variant | > 50× |
| TCGA-BT-A20T-11A-11D-A14W-08 | Bladder Urothelial Carcinoma | Broad Institute | HG19 Broad Variant | > 50× |
| TCGA-BT-A20P-11A-11D-A14W-08 | Bladder Urothelial Carcinoma | Broad Institute | HG19 Broad Variant | > 50× |
| TCGA-BT-A20Q-11A-11D-A14W-08 | Bladder Urothelial Carcinoma | Broad Institute | HG19 Broad Variant | > 50× |
| TCGA-BR-4255-11A-01D-A290-08 | Stomach adenocarcinoma | Broad Institute | HG19 Broad Variant | > 50× |
| TCGA-CG-5730-11A-01D-1600-08 | Stomach adenocarcinoma | Broad Institute | HG19 Broad Variant | > 50× |
| TCGA-EL-A3MY-11A-12D-A21A-08 | Thyroid carcinoma | Broad Institute | HG19 Broad Variant | > 50× |
| TCGA-10-0937-11A-01D-A324-10 | Ovarian serous cystadenocarcinoma | Baylor College of Medicine | GRCh37-lite | > 50× |
| TCGA-69-7763-10A-01D-A46I-10 | Lung adenocarcinoma | Baylor College of Medicine | GRCh37-lite | > 50× |
| TCGA-CA-6718-10A-01D-1835-10 | Colon adenocarcinoma | Baylor College of Medicine | GRCh37-lite | > 50× |
| TCGA-KL-8332-11A-01D-2310-10 | Kidney Chromophobe | Baylor College of Medicine | GRCh37-lite | > 50× |
| TCGA-KM-8438-10A-01D-2311-10 | Kidney Chromophobe | Baylor College of Medicine | GRCh37-lite | > 50× |
| TCGA-KO-8406-11A-01D-2311-10 | Kidney Chromophobe | Baylor College of Medicine | GRCh37-lite | > 50× |
| TCGA-NJ-A4YQ-10A-01D-A46J-10 | Lung adenocarcinoma | Baylor College of Medicine | GRCh37-lite | > 50× |
| TCGA-KM-8639-10A-01D-2397-10 | Kidney Chromophobe | Baylor College of Medicine | GRCh37-lite | > 50× |
| TCGA-KM-8477-10A-01D-2311-10 | Kidney Chromophobe | Baylor College of Medicine | GRCh37-lite | > 50× |
| TCGA-KL-8343-11A-01D-2310-10 | Kidney Chromophobe | Baylor College of Medicine | GRCh37-lite | > 50× |
| TCGA-KL-8327-11A-01D-2310-10 | Kidney Chromophobe | Baylor College of Medicine | GRCh37-lite | > 50× |
| TCGA-FV-A3I1-10A-01D-A22F-10 | Liver hepatocellular carcinoma | Baylor College of Medicine | GRCh37-lite | > 50× |
| TCGA-CC-5261-10A-01D-A12Z-10 | Liver hepatocellular carcinoma | Baylor College of Medicine | GRCh37-lite | > 50× |
| TCGA-CA-6717-10A-01D-1835-10 | Colon adenocarcinoma | Baylor College of Medicine | GRCh37-lite | > 50× |
| TCGA-B3-3926-11A-01D-1253-10 | Kidney renal papillary cell carcinoma | Baylor College of Medicine | GRCh37-lite | > 50× |

**Supplementary Table 3. HK2 integrations searched for in samples.**

| **Chromosome** | **Position** | | **Repetitive element in vicinity** | **Reference or**  **Non-reference** | **Literature** | **Status** |
| --- | --- | --- | --- | --- | --- | --- |
| 1 | 1345186 | 1346153 | MER74B | Reference | Subramanian et al. (2011) |  |
| 1 | 52471916 | 52472883 | LTR8 | Reference | Subramanian et al. (2011) |  |
| 1 | 66890497 | 66891457 | HERV9-int | Reference | Subramanian et al. (2011) |  |
| 1 | 75842771 | 75849143 | L1PA2 | Reference | Subramanian et al. (2011) | Polymorphic |
| 1 | 79792629 | 79792630 | AluSz | Non-Reference | Wildschutte et al. (2016) | Polymorphic |
| 1 | 93742338 | 93743306 |  | Reference | Subramanian et al. (2011) |  |
| 1 | 106015874 | 106015881 |  | Non-Reference | Marchi et al. (2014) | Polymorphic |
| 1 | 111802591 | 111802598 | MLT1A1 | Non-Reference | Marchi et al. (2014) | Polymorphic (Denisova) |
| 1 | 155596457 | 155605636 | HERV17-int | Reference | Subramanian et al. (2011) |  |
| 1 | 156149013 | 156149981 | AluSx1 | Reference | Subramanian et al. (2011) |  |
| 1 | 223578303 | 223578310 | L1MDa | Non-Reference | Marchi et al. (2014) | Polymorphic |
| 1 | 224527532 | 224528500 | L1M4 | Reference | Subramanian et al. (2011) |  |
| 2 | 27682845 | 27683813 |  | Reference | Subramanian et al. (2011) |  |
| 2 | 30836337 | 30837305 | L2a | Reference | Subramanian et al. (2011) |  |
| 2 | 37452460 | 37453428 | MIRb | Reference | Subramanian et al. (2011) |  |
| 2 | 130719538 | 130722209 |  | Reference | Subramanian et al. (2011) |  |
| 2 | 207901556 | 207902517 | MIRc | Reference | Subramanian et al. (2011) |  |
| 2 | 229228357 | 229229325 | L1MD | Reference | Subramanian et al. (2011) |  |
| 2 | 231708353 | 231709311 |  | Reference | Subramanian et al. (2011) |  |
| 2 | 232441494 | 232442454 |  | Reference | Subramanian et al. (2011) |  |
| 3 | 14132684 | 14133652 |  | Reference | Subramanian et al. (2011) | Polymorphic |
| 3 | 47301023 | 47301991 |  | Reference | Subramanian et al. (2011) |  |
| 3 | 50557322 | 50558279 |  | Reference | Subramanian et al. (2011) |  |
| 3 | 54011540 | 54012508 |  | Reference | Subramanian et al. (2011) |  |
| 3 | 75794210 | 75794245 |  | Non-Reference | Lee et al. (2012) | Polymorphic |
| 3 | 94943488 | 94943489 | L1PA10 | Non-Reference | Wildschutte et al. (2016) | Polymorphic |
| 3 | 112743479 | 112752282 | MLT2C1 | Reference | Subramanian et al. (2011) | Polymorphic |
| 3 | 125609302 | 125618416 | AluYa8 | Reference | Subramanian et al. (2011) |  |
| 3 | 129360117 | 129360905 |  | Reference | Subramanian et al. (2011) |  |
| 3 | 175623337 | 175624305 | L1PA5 | Reference | Subramanian et al. (2011) |  |
| 3 | 185280336 | 185289515 |  | Reference | Subramanian et al. (2011) |  |
| 3 | 186611008 | 186611975 | MLT1B | Reference | Subramanian et al. (2011) |  |
| 3 | 195654395 | 195655363 | L1PB4 | Reference | Subramanian et al. (2011) |  |
| 3 | 197845799 | 197845879 | PRIMA41-int | Non-Reference | Lee et al. (2012) | Polymorphic |
| 4 | 145520 | 146488 | AluSq | Reference | Subramanian et al. (2011) |  |
| 4 | 9603239 | 9603245 | HERVS71-int | Non-Reference | Marchi et al. (2014) | Polymorphic |
| 4 | 9981605 | 9981606 | L2b | Non-Reference | Wildschutte et al. (2016) | Polymorphic |
| 4 | 63806595 | 63807563 | LTR81B | Reference | Subramanian et al. (2011) |  |
| 4 | 72994940 | 72995908 | L2a | Reference | Subramanian et al. (2011) |  |
| 4 | 120263688 | 120264654 | AluSx | Reference | Subramanian et al. (2011) |  |
| 4 | 157225913 | 157226870 |  | Reference | Subramanian et al. (2011) |  |
| 4 | 165826665 | 165827635 |  | Reference | Subramanian et al. (2011) |  |
| 4 | 190966833 | 190966919 | LTR43-int | Non-Reference | Lee et al. (2012) | Polymorphic |
| 5 | 1596091 | 1597059 |  | Reference | Subramanian et al. (2011) |  |
| 5 | 4537604 | 4537605 | LTR1C | Non-Reference | Wildschutte et al. (2016) | Polymorphic |
| 5 | 8937853 | 8938820 | L1PA13 | Reference | Subramanian et al. (2011) |  |
| 5 | 35176486 | 35177455 | L1MB7 | Reference | Subramanian et al. (2011) |  |
| 5 | 44730588 | 44731556 | L1PA8 | Reference | Subramanian et al. (2011) |  |
| 5 | 54866907 | 54867867 |  | Reference | Subramanian et al. (2011) |  |
| 5 | 64388439 | 64388446 | L1M6 | Non-Reference | Marchi et al. (2014) | Polymorphic (Neanderthal) |
| 5 | 74901657 | 74902617 | L1MEd | Reference | Subramanian et al. (2011) |  |
| 5 | 80442265 | 80442272 |  | Non-Reference | Marchi et al. (2014) | Polymorphic (Denisova & Neanderthal) |
| 5 | 116156834 | 116157794 | AluSx1 | Reference | Subramanian et al. (2011) |  |
| 5 | 156084717 | 156093896 | AluSx | Reference | Subramanian et al. (2011) |  |
| 5 | 169423878 | 169424838 | AluYh9 | Reference | Subramanian et al. (2011) |  |
| 5 | 178940963 | 178941931 | L1ME3A | Reference | Subramanian et al. (2011) |  |
| 6 | 16004793 | 16004926 |  | Non-Reference | Wildschutte et al. (2016) | Polymorphic |
| 6 | 27742238 | 27743207 |  | Reference | Subramanian et al. (2011) |  |
| 6 | 32643383 | 32643537 | L1PA10 | Non-Reference |  | Polymorphic |
| 6 | 32648035 | 32648041 | L1PA10 | Non-Reference | Marchi et al. (2014) | Polymorphic |
| 6 | 33777736 | 33778696 |  | Reference | Subramanian et al. (2011) |  |
| 6 | 34687236 | 34688205 |  | Reference | Subramanian et al. (2011) |  |
| 6 | 52787477 | 52788438 | HERVE-int | Reference | Subramanian et al. (2011) | Polymorphic |
| 6 | 78427019 | 78436083 |  | Reference | Subramanian et al. (2011) | Polymorphic |
| 6 | 79568504 | 79569472 | AluSx | Reference | Subramanian et al. (2011) |  |
| 6 | 89091306 | 89092274 |  | Reference | Subramanian et al. (2011) |  |
| 6 | 93883083 | 93884048 | MLT1B | Reference | Subramanian et al. (2011) |  |
| 6 | 111576299 | 111577258 |  | Reference | Subramanian et al. (2011) |  |
| 6 | 134980247 | 134981215 |  | Reference | Subramanian et al. (2011) |  |
| 6 | 151776344 | 151777320 |  | Reference | Subramanian et al. (2011) |  |
| 6 | 161270898 | 161270905 |  | Non-Reference | Marchi et al. (2014) | Polymorphic (Denisova) |
| 7 | 4622057 | 4640031 |  | Reference | Subramanian et al. (2011) | Polymorphic |
| 7 | 4630561 | 4640031 | AluSx | Reference | Subramanian et al. (2011) | Polymorphic |
| 7 | 16237346 | 16238314 | MER67B | Reference | Subramanian et al. (2011) |  |
| 7 | 23079474 | 23080442 | L1PA2 | Reference | Subramanian et al. (2011) |  |
| 7 | 104388369 | 104393266 | L1MC2 | Reference | Subramanian et al. (2011) |  |
| 7 | 123420690 | 123421658 |  | Reference | Subramanian et al. (2011) |  |
| 7 | 124861223 | 124862191 |  | Reference | Subramanian et al. (2011) |  |
| 7 | 125808213 | 125809181 |  | Reference | Subramanian et al. (2011) |  |
| 7 | 158029482 | 158030450 |  | Reference | Subramanian et al. (2011) |  |
| 7 | 158773312 | 158773459 |  | Non-Reference | Wildschutte et al. (2016) | Polymorphic |
| 8 | 7355397 | 7364859 | MIR | Reference | Subramanian et al. (2011) | Polymorphic |
| 8 | 18651458 | 18652426 |  | Reference | Subramanian et al. (2011) | Polymorphic |
| 8 | 37050885 | 37051853 |  | Reference | Subramanian et al. (2011) |  |
| 8 | 43594681 | 43595649 |  | Reference | Subramanian et al. (2011) |  |
| 8 | 48087655 | 48088615 | AluSx | Reference | Subramanian et al. (2011) |  |
| 8 | 54944240 | 54945208 | L1MB5 | Reference | Subramanian et al. (2011) |  |
| 8 | 58112161 | 58113123 |  | Reference | Subramanian et al. (2011) |  |
| 8 | 91696259 | 91697227 |  | Reference | Subramanian et al. (2011) |  |
| 8 | 140472149 | 140475236 |  | Reference | Subramanian et al. (2011) |  |
| 8 | 144914158 | 144915127 | HAL1 | Reference | Subramanian et al. (2011) |  |
| 8 | 146021278 | 146022246 |  | Reference | Subramanian et al. (2011) |  |
| 8 | 146086169 | 146086170 |  | Non-Reference | Wildschutte et al. (2016) | Polymorphic |
| 9 | 17445358 | 17446326 |  | Reference | Subramanian et al. (2011) |  |
| 9 | 31632876 | 31633844 |  | Reference | Subramanian et al. (2011) |  |
| 9 | 68277342 | 68278302 | AluSx | Reference | Subramanian et al. (2011) |  |
| 9 | 72378052 | 72379020 |  | Reference | Subramanian et al. (2011) |  |
| 9 | 111356892 | 111357852 | MIR | Reference | Subramanian et al. (2011) |  |
| 9 | 124191383 | 124192351 |  | Reference | Subramanian et al. (2011) |  |
| 9 | 132205208 | 132205208 | MLT1C | Non-Reference | Lee et al. (2012) | Polymorphic |
| 9 | 134232105 | 134233063 |  | Reference | Subramanian et al. (2011) |  |
| 9 | 136957557 | 136958526 | AluJb | Reference | Subramanian et al. (2011) |  |
| 10 | 27182399 | 27183380 | MER34-int | Reference | Subramanian et al. (2011) | Polymorphic |
| 10 | 42526055 | 42527021 |  | Reference | Subramanian et al. (2011) |  |
| 10 | 43832650 | 43833618 |  | Reference | Subramanian et al. (2011) |  |
| 10 | 70285428 | 70286393 |  | Reference | Subramanian et al. (2011) |  |
| 10 | 101016044 | 101016228 | MSTD | Non-Reference |  | Polymorphic |
| 10 | 101580569 | 101587716 |  | Reference | Subramanian et al. (2011) | Polymorphic |
| 10 | 134444012 | 134444013 | LTR46 | Non-Reference | Wildschutte et al. (2016) | Polymorphic |
| 11 | 10412855 | 10413823 | L2b | Reference | Subramanian et al. (2011) |  |
| 11 | 24467639 | 24468598 | MLT1F1 | Reference | Subramanian et al. (2011) |  |
| 11 | 60449889 | 60449889 | L1M4 | Non-Reference | Marchi et al. (2014) | Polymorphic (Denisova) |
| 11 | 61422484 | 61423448 |  | Reference | Subramanian et al. (2011) |  |
| 11 | 61962131 | 61963100 | MLT1D | Reference | Subramanian et al. (2011) |  |
| 11 | 63295442 | 63296410 | MER21B | Reference | Subramanian et al. (2011) |  |
| 11 | 63297785 | 63298753 |  | Reference | Subramanian et al. (2011) |  |
| 11 | 67370824 | 67371784 | LTR26 | Reference | Subramanian et al. (2011) |  |
| 11 | 67635434 | 67636394 | L1PA4 | Reference | Subramanian et al. (2011) |  |
| 11 | 71875417 | 71876385 | MER61-int | Reference | Subramanian et al. (2011) |  |
| 11 | 101565794 | 101575259 |  | Reference | Subramanian et al. (2011) | Polymorphic |
| 12 | 6995023 | 6995985 |  | Reference | Subramanian et al. (2011) |  |
| 12 | 9753060 | 9754028 |  | Reference | Subramanian et al. (2011) |  |
| 12 | 30084986 | 30085954 |  | Reference | Subramanian et al. (2011) |  |
| 12 | 32252444 | 32253412 | L1MB8 | Reference | Subramanian et al. (2011) |  |
| 12 | 44313656 | 44313662 | L1MB1 | Non-Reference | Marchi et al. (2014) | Polymorphic (Neanderthal) |
| 12 | 51848070 | 51849038 | AluJb | Reference | Subramanian et al. (2011) |  |
| 12 | 55727214 | 55728183 |  | Reference | Subramanian et al. (2011) | Polymorphic |
| 12 | 56794144 | 56795109 | HERVL-int | Reference | Subramanian et al. (2011) |  |
| 12 | 58721242 | 58730698 | L2b | Reference | Subramanian et al. (2011) | Polymorphic |
| 12 | 105872336 | 105873305 |  | Reference | Subramanian et al. (2011) |  |
| 12 | 111007843 | 111009325 |  | Reference | Subramanian et al. (2011) |  |
| 12 | 118543788 | 118544748 | AluY | Reference | Subramanian et al. (2011) |  |
| 12 | 124066476 | 124066483 | AluSx1 | Non-Reference | Marchi et al. (2014) | Polymorphic |
| 13 | 20174357 | 20175325 |  | Reference | Subramanian et al. (2011) |  |
| 13 | 50173157 | 50174126 |  | Reference | Subramanian et al. (2011) |  |
| 13 | 90743182 | 90743189 |  | Non-Reference | Marchi et al. (2014) | Polymorphic (Neanderthal) |
| 14 | 55491267 | 55492221 | L2c | Reference | Subramanian et al. (2011) |  |
| 14 | 65445305 | 65446273 | LTR107_Mam | Reference | Subramanian et al. (2011) |  |
| 15 | 28430044 | 28430186 |  | Non-Reference | Lee et al. (2012); Wildschutte et al. (2016) | Polymorphic |
| 15 | 63374593 | 63374600 | AluJr | Non-Reference | Marchi et al. (2014) | Polymorphic |
| 15 | 65518879 | 65519847 |  | Reference | Subramanian et al. (2011) |  |
| 15 | 89083782 | 89084750 | HERVS71-int | Reference | Subramanian et al. (2011) |  |
| 15 | 102400179 | 102401147 | LTR64 | Reference | Subramanian et al. (2011) |  |
| 16 | 5804388 | 5805356 |  | Reference | Subramanian et al. (2011) |  |
| 16 | 8228762 | 8229730 |  | Reference | Subramanian et al. (2011) |  |
| 16 | 23610804 | 23611764 | L2c | Reference | Subramanian et al. (2011) |  |
| 16 | 47898369 | 47899337 |  | Reference | Subramanian et al. (2011) |  |
| 16 | 74833301 | 74834258 | L1MB3 | Reference | Subramanian et al. (2011) |  |
| 17 | 4977974 | 4978941 |  | Reference | Subramanian et al. (2011) |  |
| 17 | 29027058 | 29028026 | AluSx | Reference | Subramanian et al. (2011) |  |
| 17 | 52278934 | 52279902 | L1ME4b | Reference | Subramanian et al. (2011) |  |
| 17 | 66600132 | 66601092 | L2b | Reference | Subramanian et al. (2011) |  |
| 17 | 78525007 | 78525976 | L2a | Reference | Subramanian et al. (2011) |  |
| 18 | 2000814 | 2001782 | HAL1 | Reference | Subramanian et al. (2011) |  |
| 18 | 4917277 | 4918246 | THE1B-int | Reference | Subramanian et al. (2011) |  |
| 19 | 21841536 | 21841542 |  | Non-Reference | Marchi et al. (2014) | Polymorphic (Denisova) |
| 19 | 22414303 | 22414381 | L1M3 | Non-Reference | Lee et al. (2012) | Polymorphic |
| 19 | 22457244 | 22457245 | AluSq | Non-Reference | Wildschutte et al. (2016) | Polymorphic (Denisova) |
| 19 | 28128498 | 28137361 | AluYa5 | Reference | Subramanian et al. (2011) |  |
| 19 | 29855781 | 29855787 |  | Non-Reference | Marchi et al. (2014) | Polymorphic (Denisova) |
| 19 | 36738045 | 36738826 | AluSx | Reference | Subramanian et al. (2011) |  |
| 19 | 37822292 | 37823274 | AluSq2 | Reference | Subramanian et al. (2011) |  |
| 19 | 38120851 | 38121813 | L1ME3A | Reference | Subramanian et al. (2011) |  |
| 19 | 38356816 | 38357784 | AluJb | Reference | Subramanian et al. (2011) |  |
| 19 | 45097417 | 45098385 |  | Reference | Subramanian et al. (2011) |  |
| 19 | 52546384 | 52547353 |  | Reference | Subramanian et al. (2011) |  |
| 19 | 57996939 | 57996940 |  | Non-Reference | Wildschutte et al. (2016) | Polymorphic (Neanderthal) |
| 20 | 12402386 | 12402392 |  | Non-Reference | Lee et al. (2012) & Marchi et al. (2014) | Polymorphic (Denisova) |
| 20 | 33849926 | 33850894 | L2c | Reference | Subramanian et al. (2011) |  |
| 20 | 40599535 | 40600503 | L2a | Reference | Subramanian et al. (2011) |  |
| 21 | 19933916 | 19941962 |  | Reference | Subramanian et al. (2011) |  |
| 21 | 44567239 | 44568199 |  | Reference | Subramanian et al. (2011) |  |
| 22 | 18926187 | 18935307 | AluJb | Reference | Subramanian et al. (2011) |  |
| 22 | 23852639 | 23852640 | MLT1C | Non-Reference | Wildschutte et al. (2016) | Polymorphic |
| X | 90205613 | 90206582 | LTR17 | Reference | Subramanian et al. (2011) |  |
| X | 93606603 | 93606604 | L1MD1 | Non-Reference | Wildschutte et al. (2016) | Polymorphic (Denisova) |
| X | 124913492 | 124914460 | L1PB | Reference | Subramanian et al. (2011) |  |
| X | 144802358 | 144803318 |  | Reference | Subramanian et al. (2011) |  |
| Y | 6616929 | 6617896 | L1PA10 | Reference | Subramanian et al. (2011) |  |
| Y | 15215736 | 15216703 | L1PB4 | Reference | Subramanian et al. (2011) |  |
| Y | 25039185 | 25040153 |  | Reference | Subramanian et al. (2011) |  |
| Y | 26672934 | 26673902 |  | Reference | Subramanian et al. (2011) |  |
| Y | 27288494 | 27289462 |  | Reference | Subramanian et al. (2011) |  |

Greyed out cells were not included in analysis to not introduce gender bias into evaluation of polymorphisms.

**Supplementary Table 4. HK2 LTRs manually inserted into hg19 chromosome 20.**

| **Chromosome** | **Position** |
| --- | --- |
| 20 | 126699 |
| 20 | 162699 |
| 20 | 402099 |
| 20 | 410543 |
| 20 | 413551 |
| 20 | 512092 |
| 20 | 544052 |
| 20 | 572945 |
| 20 | 588355 |
| 20 | 639693 |

**Note 2**

**Technical considerations: Repetitive regions and pseudogenes**

In addition to using *STEAK* for marking presence/absence of unfixed HK2 integrations, we were also able to recover an integration within the germline of a lung adenocarcinoma patient (Figure S2) that was not picked up by the other pipelines we benchmarked. This integration is particularly difficult to recover because its exact genomic location remains obscure. Originally in our analyses, reads belonging to this integration had mapped discordantly to different chromosomes. After processing with *STEAK*, a one-sided cluster, representing host reads flanking only one end of the LTR, was recovered in chromosome 3 in an exon of pseudogene FAM86DP (Figure 5A)*.*

Eukaryotic elongation factor2 lysine methyltransferases (FAM86) is a family of genes and pseudogenes, which are dispersed throughout the human genome. Because of the various FAM86 copies, the cluster of reads prior to trimming had originally been mapped to different locations. By using the breakpoint interval observed with the trimmed reads produced by *STEAK*, we were able to create a contig of the unknown left flank of the HK2 integration and recover the original site. We produced a junction of the opposite side of the LTR with a predicted 4 bp target site duplication (TSD) and the rest of the exon. By remapping all reads against this contig, we were able to extract the reads that belonged to the other side of the HK2 integration and observed a 5 bp TSD of “CTTGG” (Figure S2B). While we were able to reconstruct the pre-integration site, we were still unable to confidently assign this integration a genomic location. Using BLAT we compared the pre-integration sequence against the hg19 genome. The top results with the highest percent identities (≤ 96.9%) consisted of FAM86 copies. While this almost 4% divergence between our pre-integration site and a particular FAM86 gene or pseudogene leaves the precise genomic location of our novel integration unidentified, we can confirm that this particular integration is embedded in a FAM86-related gene or pseudogene.


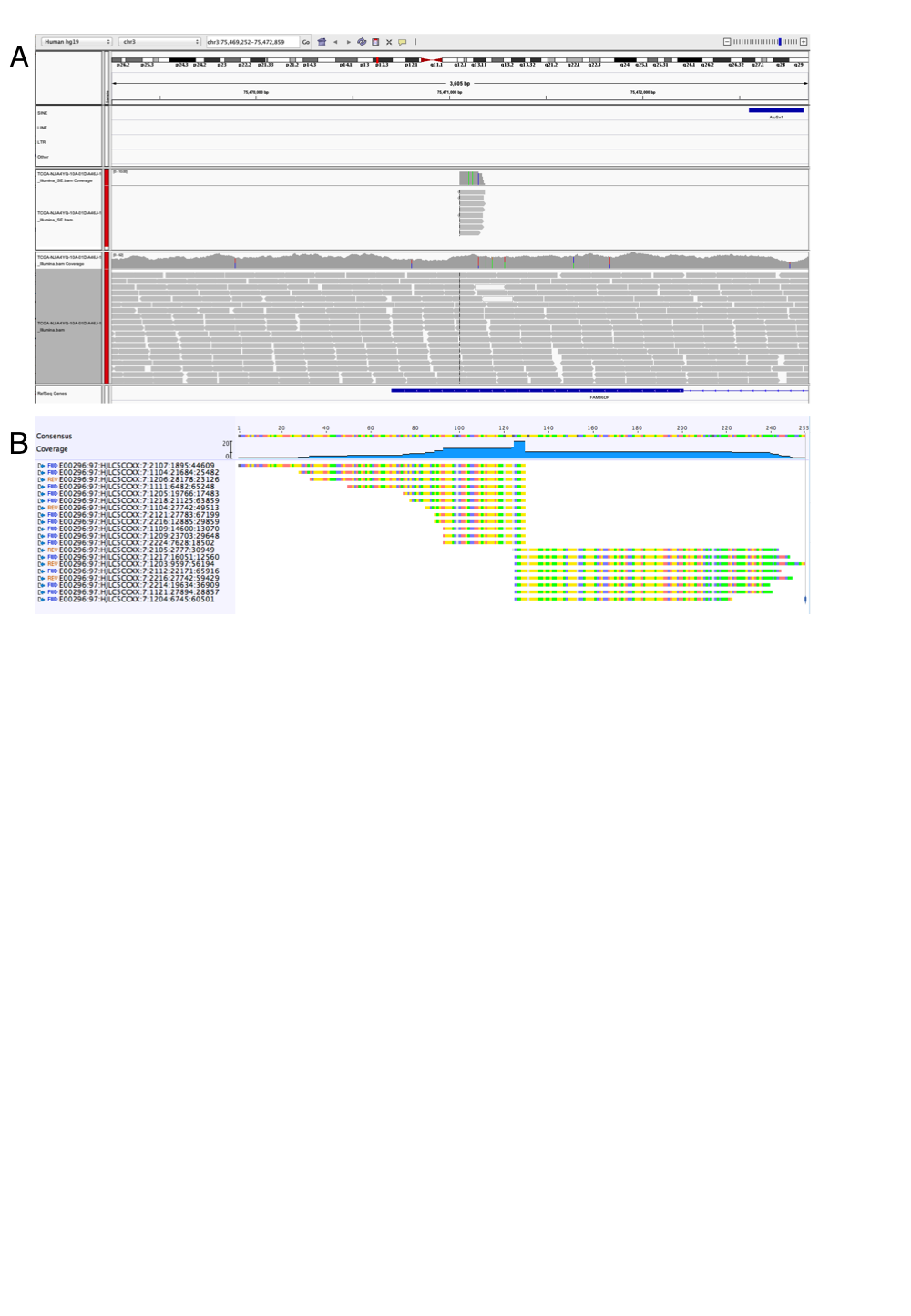


Supplementary Figure 1. The detection of a novel integration in a non-reference pseudogene/gene. (A) IGV screenshot of a one-sided cluster of trimmed reads in the exon of FAM86DP. (B) *De novo* reconstruction of preintegration site using trimmed reads. The peak of coverage displays the TSD, characteristic of retroviral integration.

### Note 3: Pilot performance on simulated genomes

We had previously evaluated *STEAK’s* performance by testing two simulated paired-end NGS datasets. The first dataset consists of reads made from human chromosome 20 with artificial AluY insertions at 5X coverage originally created by *Tangram*. The second dataset is also derived from chromosome 20 but with artificial HK2 integrations and at 50X coverage. This second dataset was created to evaluate the performance in a high coverage genome and to benchmark against *VirusSeq*. However, on our simulated dataset we noted that no HK2 integrations were picked up on *VirusSeq.* As a test to confirm *VirusSeq* was truly not finding HK2 and that it was not a troubleshooting error, we created another simulated genome at 50X coverage with the same HK2 insertions with an additional HBV insertion. After running *VirusSeq* on this simulated genome, we observed that *VirusSeq* was only able to recognise the HBV integration but none of the HK2 integrations. This suggests that *VirusSeq* works best in finding viruses that are not found at all in the host reference.

*Tangram* was the most sensitive of the three programs performing on the 5X coverage genome. However, *STEAK* maintained 65% positive predictive value (PPV which is the probability that a suggested integration is true), whereas *RetroSeq* and *Tangram* achieved PPVs below 50% (Supplementary Figure 2A). We evaluated specificity prior to filtering for known insertions since both *RetroSeq* and *Tangram* are software that aim to search for non-reference putative insertions. We ran *STEAK* with parameters to search for non-reference insertions only. PPVs were calculated by comparing the number of true positive calls to the total number of positive calls (including false positives) when marking the simulated genomes.

On the 50X coverage dataset, *STEAK* gave no false positives compared to *RetroSeq* (Supplementary Figure 2B). However, two of the insertions within other repetitive elements, such as LINEs and SINEs, could not be recovered by *STEAK* whereas *RetroSeq* was able to do so (Supplementary Figure 2B). *VirusSeq* could not recover any HK2 integrations.


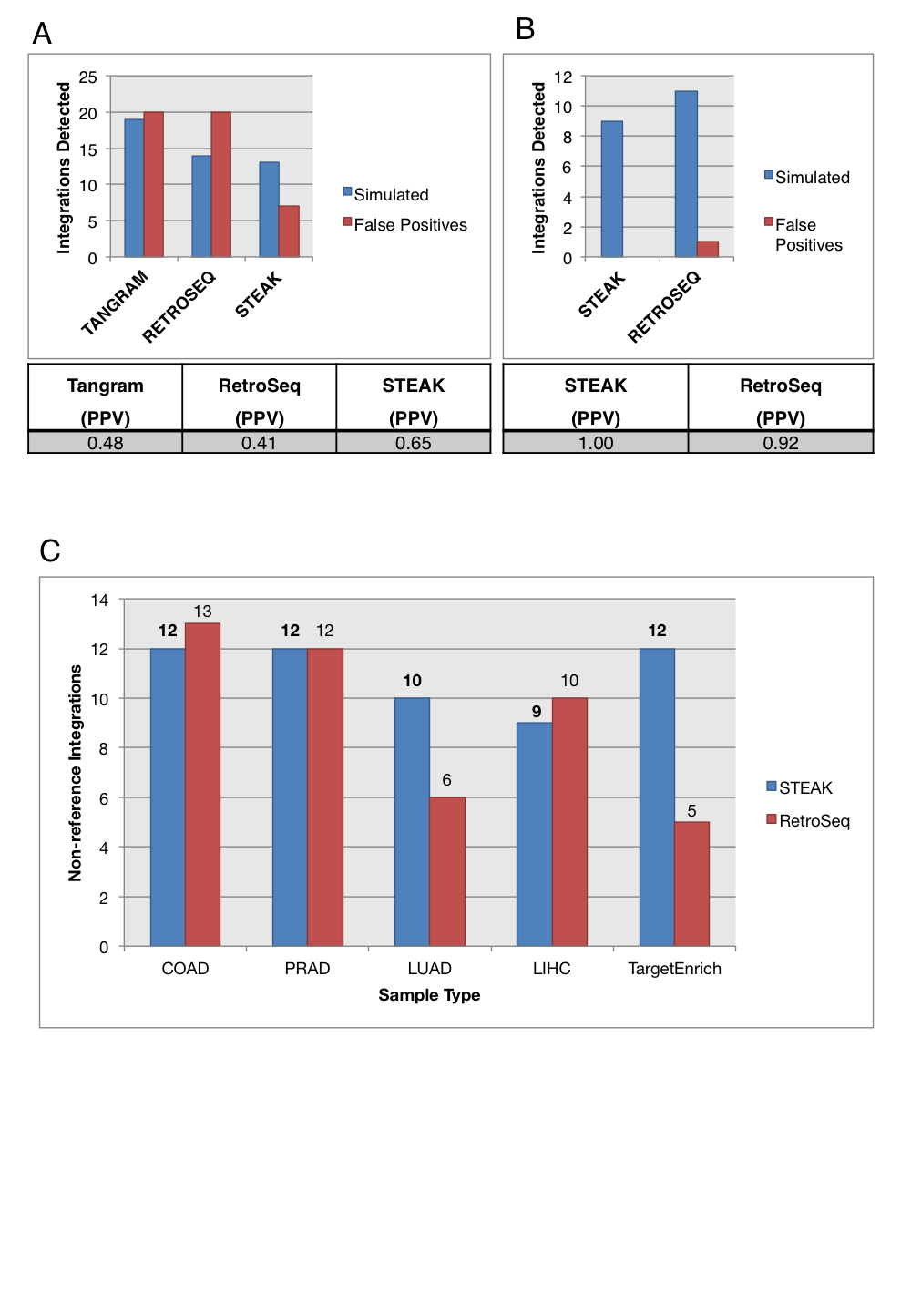


Supplementary Figure 2. Comparative performance of *Tangram, RetroSeq, and STEAK* on simulated data. (A&B) Blue indicates detection of simulated integrations and red indicated the number of false positive detected by each respective program. (A) Bar graph shows integrations detected in 5x coverage simulated genome with 20 artificial Alu insertions. The table below gives the positive predictive value (PPV) for evaluation of specificity. (B) Integrations detected in a 50x coverage simulated genome with 11 artificial HK2 integrations. The tables below A & B give the PPV for each program tested.

### Note 4: Commands used for analyses and benchmarking

Tangram

-Simulation data script provided by Tangram works with their files from their repository (e.g. 5X coverage ALU simulation).

-Below are the commands used on real WGS data:

tangram_bam -i NA12878.platinum.bwa_merge.bam -r HK113LTR.fasta -o NA12878_tangram.bam

tangram_index -ref hs37d5.fasta -sp HK113LTR.fasta -out hs37d5_tangram.ref

tangram_scan -in tangram_scan.list -dir ./Tangram/

tangram_detect -lb ./Tangram/output_tangram/lib_table.dat //

-ht./Tangram/output_tangram/hist.dat -in tangram_bam.list -rg 20 //

-ref hs37d5_tangram.ref

##With MOSAIK aligner BAM:

MosaikBuild -fr hs37d5.fa.gz -oa hs37d5.bin

MosaikBuild -q NA12878_PE.fastq -out tangram_NA12878_PE.bin -st illumina

MosaikJump -ia hs37d5.bin -out hs37d5.jmp -hs 15

MosaikBuild -fr HK113LTR.fasta -oa HK113LTR.bin

MosaikJump -ia HK113LTR.bin -out HK113LTR.jmp -hs 15

MosaikAligner -ia hs37d5.bin -j hs37d5.jmp -sref HK113LTR -srefn 50 -annpe ./MOSAIK/src/networkFile/2.1.26.pe.100.0065.ann -annse ./MOSAIK/src/networkFile/2.1.26.se.100.005.ann -mmp 0.15 -mhp 200 -ls 476 -act 20 -bw 29 -p 8 -in tangram_NA12878_PE.bin -out tangram_NA12878_PE.bam

(Both end up in a Segmentation Fault when put through tangram_detect. Tangram was installed locally as well as a module on ARC. Both installations led to the same error.)

VirusSeq for 50x simulation with HK2

gzip COAD.1.fastq
gzip COAD.2.fastq

MosaikBuild -q $DATA/benchmark/COAD/COAD.1.fastq.gz -q2 $DATA/benchmark/COAD/COAD.2.fastq.gz -out $DATA/benchmark/COAD/COAD_Virus.bin -st illumina

MosaikAligner -in $DATA/benchmark/COAD/COAD_Virus.bin -ia $DATA/benchmark/virusseq/jumpdb/hg19.fa.bin -out $DATA/benchmark/COAD/COAD.bin.aligned -hs 15 -mmp 0.1 -mmal -minp 0.5 -act 25 -mhp 100 -m unique -j $DATA/benchmark/virusseq/jumpdb/hg19.JumpDb -p 16 -km -pm -rur $DATA/benchmark/COAD/COAD_unalg.fq

MosaikJump -ia hg19Virus.fa.bin -out hg19Virus.JumpDb -hs 15 -mhp 100 -km

MosaikJump -ia hg19.fa.bin -out hg19.JumpDb -hs 15 -mhp 100 -km

MosaikJump -ia $DATA/benchmark/virusseq/jumpdb/hg19Virus.bin -out $DATA/benchmark/virusseq/jumpdb/hg19Virus.jmp -hs 15 -mhp 100 -km

##for gib virus reference genome

MosaikBuild -fr $DATA/tools/VirusSeq/gibVirus.fa -oa $DATA/tools/VirusSeq/gibVirus.fa.bin -st illumina -assignQual 40

MosaikJump -ia $DATA/tools/VirusSeq/gibVirus.fa.bin -out $DATA/tools/VirusSeq/gibVirus.JumpDb -hs 15 -mhp 100 -km

##version-0.89 and for combined hg19Virus reference genome

MosaikBuild -fr $DATA/benchmark/virusseq/jumpdb/hg19Virus.fa -oa $DATA/benchmark/virusseq/jumpdb/hg19Virus.fa.bin -st illumina -assignQual 40

MosaikJump -ia $DATA/benchmark/virusseq/jumpdb/hg19Virus.fa.bin -out $DATA/benchmark/virusseq/jumpdb/hg19Virus.JumpDb -hs 15 -mhp 100 -km

##for hg19 reference genome

MosaikBuild -fr $DATA/references/hg19/hg19.fa -oa hg19.fa.bin -st illumina -assignQual 40

MosaikJump -ia hg19.fa.bin -out hg19.JumpDb -hs 15 -mhp 100 –km

MosaikAligner -in $DATA/benchmark/simulated/750K/sim_Virus.bin -ia MosaikBuild -q $DATA/benchmark/simulated/750K/HBV_HK2_sim_1.fq -out $DATA/benchmark/simulated/750K/sim50x_1.bin -st illumina

MosaikBuild -q $DATA/benchmark/simulated/750K/HBV_HK2_sim_2.fq -out $DATA/benchmark/simulated/750K/sim50x_2.bin -st illumina

MosaikAligner -in $DATA/benchmark/simulated/750K/sim50x_1.bin -ia $DATA/benchmark/virusseq/jumpdb/hg19Virus.fa.bin -out $DATA/benchmark/simulated/750K/sim50x_1.bin.aligned -hs 15 -mmp 0.1 -mmal -minp 0.5 -act 20 -mhp 100 -m unique -j $DATA/benchmark/virusseq/jumpdb/hg19Virus.JumpDb -p 16 -km -pm

MosaikAligner -in $DATA/benchmark/simulated/750K/sim50x_2.bin -ia $DATA/benchmark/virusseq/jumpdb/hg19Virus.fa.bin -out $DATA/benchmark/simulated/750K/sim50x_2.bin.aligned -hs 15 -mmp 0.1 -mmal -minp 0.5 -act 20 -mhp 100 -m unique -j $DATA/benchmark/virusseq/jumpdb/hg19Virus.JumpDb -p 16 -km -pm

MosaikSort -in $DATA/benchmark/simulated/750K/sim50x_1.bin.aligned -out $DATA/benchmark/simulated/750K/sim50x_1.bin.sorted

MosaikSort -in $DATA/benchmark/simulated/750K/sim50x_2.bin.aligned -out $DATA/benchmark/simulated/750K/sim50x_2.bin.sorted

MosaikMerge -in $DATA/benchmark/simulated/750K/sim50x_1.bin.sorted -in $DATA/benchmark/simulated/750K/sim50x_2.bin.sorted -out $DATA/benchmark/simulated/750K/sim50x_SE.sorted

MosaikText -in $DATA/benchmark/simulated/750K/sim50x_SE.sorted -axt $DATA/benchmark/simulated/750K/sim50x_SE.sorted.axt

MosaikAligner -in $DATA/benchmark/simulated/750K/sim50x.bin -ia $DATA/benchmark/virusseq/jumpdb/hg19.fa.bin -out $DATA/benchmark/simulated/750K/sim50x.bin.aligned -hs 15 -mmp 0.1 -mmal -minp 0.5 -act 25 -mhp 100 -m unique -j $DATA/benchmark/virusseq/jumpdb/hg19.JumpDb -p 16 -km -pm -rur $DATA/benchmark/simulated/750K/sim_unalg.fq

MosaikBuild -q $DATA/benchmark/simulated/750K/sim_unalg.fq -out $DATA/benchmark/simulated/750K/sim_Virus.bin -st illumina

MosaikAligner -in $DATA/benchmark/simulated/750K/sim_Virus.bin -ia $DATA/benchmark/virusseq/jumpdb/gibVirus.bin -out $DATA/benchmark/simulated/750K/sim_Virus.bin.aligned -hs 15 -mmp 0.10 -act 20 -mhp 100 -m all -j $DATA/benchmark/virusseq/jumpdb/gibVirus.jmp -p 16 -km -pm

MosaikSort -in $DATA/benchmark/simulated/750K/sim_Virus.bin.aligned -out $DATA/benchmark/simulated/750K/sim_Virus.bin.aligned.sorted

MosaikAssembler -in $DATA/benchmark/simulated/750K/sim_Virus.bin.aligned.sorted -ia $DATA/benchmark/virusseq/jumpdb/gibVirus.bin -out $DATA/benchmark/simulated/750K/sim_Virus.bin.assembled -f ace > $DATA/benchmark/simulated/750K/sim_viruslog.txt

perl $DATA/benchmark/virusseq/scripts/VirusSeq_Detection.pl $DATA/benchmark/simulated/750K/sim_viruslog.txt 1000 $DATA/benchmark/simulated/750K/sim_virusName.txt

Spanner --scan --infile $DATA/benchmark/simulated/750K/sim50x.bin.viral --outdir $DATA/benchmark/simulated/750K/SV_gDNA

Spanner --build --infile $DATA/benchmark/simulated/750K/sim50x.bin.viral --outdir $DATA/benchmark/simulated/750K/SV_gDNA -f $DATA/benchmark/simulated/750K/SV_gDNA/MSK.stats -a $DATA/benchmark/virusseq/jumpdb/Spanner_anchor_hg19Virus.txt -t

perl $DATA/benchmark/simulated/750K/SV_gDNA/Spanner_cross_converter.pl $DATA/benchmark/virusseq/jumpdb/hg19Virus_refGene_RIS.txt $DATA/benchmark/simulated/750K/sim50x_SE.sorted.axt $DATA/benchmark/simulated/750K/sim50x_crossReads.txt

RetroSeq – Commands & parameters used for WGS and TE

retroseq.pl -discover -bam ${NAME}.bam –id 90 –len 20 -eref HK2_retroseq.list -align -output ${OUT}_retroseq

retroseq.pl -call -bam ${NAME}.bam -input ${OUT}_retroseq -ref ${REF} -output ${OUT}_retroseq.vcf

MELT – Commands & parameters

java -jar ./MELTv2.0.2/MELT.jar BuildTransposonZIP HK113LTR.fa HK2_Hs_reference.bed HK2 10

java -jar ./MELTv2.0.2/MELT.jar Single –nocleanup -l $DATA/benchmark/melt/${SAMPLE}/${SAMPLE}_h37d5_merged.sorted.bam -c 30 -h $DATA/benchmark/melt/hs37d5.fa -n ./MELTv2.0.2/add_bed_files/1KGP_Hg19/hg19.genes.bed -w $DATA/benchmark/melt/${SAMPLE}/ -t $DATA/benchmark/melt/ERVK_MELT.zip

java -jar ./MELTv2.0.2/MELT.jar Deletion-Genotype -b $DATA/benchmark/melt/HK2_Hs_reference.bed -l $DATA/benchmark/melt/${SAMPLE}/${SAMPLE}_h37d5_merged.sorted.bam -h $DATA/benchmark/melt/hs37d5.fa -w $DATA/benchmark/melt/${SAMPLE}

####For target enrichment####
java -jar ./MELTv2.0.2/MELT.jar Single –r 300 –e 1200 –nocleanup -l $DATA/benchmark/melt/${SAMPLE}/${SAMPLE}_h37d5_merged.sorted.bam -c 30 -h $DATA/benchmark/melt/hs37d5.fa -n ./MELTv2.0.2/add_bed_files/1KGP_Hg19/hg19.genes.bed -w $DATA/benchmark/melt/${SAMPLE}/ -t $DATA/benchmark/melt/ERVK_MELT.zip

java -jar ./MELTv2.0.2/MELT.jar Deletion-Genotype –e 1200 -b $DATA/benchmark/melt/HK2_Hs_reference.bed -l $DATA/benchmark/melt/${SAMPLE}/${SAMPLE}_h37d5_merged.sorted.bam -h $DATA/benchmark/melt/hs37d5.fa -w $DATA/benchmark/melt/${SAMPLE}

Tlex2 – Over 5 days and did not complete requires more than 256 GB of RAM for high coverage genomes
tlex-open-v2.pl -s human -id 90 -limp 20 -lima 20 -processes 16 -noclean -pairends yes -T ./ERVK_id.list -M ./ERVK_unique_strand.bed -G ./hs37d5.fa -R ./pedigree/NA12878/

VFS – Over 5 days and did not complete. At day 5, CAP3 phase began.
viral.fusion.pl --config vfs.conf --insertSIZE NA NA12891 ERR194160_1.fastq.gz ERR194160_2.fastq.gz

STEAK – parameters used for both WGS and TE
steak --input NA12878.PE.bam --paired --TE-reference HK113LTR.fa --alignment-quality 1.0 --match-quality 0.9 --aligned --transposon-length 20 --host-length 20Citations

Chen YX, Yao H, Thompson EJ, Tannir NM, Weinstein JN, Su XP. 2013. VirusSeq: software to identify viruses and their integration sites using next-generation sequencing of human cancer tissue. *Bioinformatics* **29**: 266-267.

Fiston-Lavier AS, Barron MG, Petrov DA, Gonzalez J. 2015. T-lex2: genotyping, frequency estimation and re-annotation of transposable elements using single or pooled next-generation sequencing data. *Nucleic Acids Research* **43**.

GitHub. 2015. Jitterbug. Available from: https://github.com/elzbth/jitterbug [Accessed 4 January 2017]

Helman E, Lawrence MS, Stewart C, Sougnez C, Getz G, Meyerson M. 2014. Somatic retrotransposition in human cancer revealed by whole-genome and exome sequencing. *Genome Res* **24**: 1053-1063.

Henaff E, Zapata L, Casacuberta JM, Ossowski S. 2015. Jitterbug: somatic and germline transposon insertion detection at single-nucleotide resolution. *Bmc Genomics* **16**.

Keane TM, Wong K, Adams DJ. 2013. RetroSeq: transposable element discovery from next-generation sequencing data. *Bioinformatics* **29**: 389-390.

Lee E, Iskow R, Yang L, Gokcumen O, Haseley P, Luquette LJ, Lohr JG, Harris CC, Ding L, Wilson RK et al. 2012. Landscape of Somatic Retrotransposition in Human Cancers. *Science* **337**: 967-971.

Lee WP, Stromberg MP, Ward A, Stewart C, Garrison EP, Marth GT. 2014. MOSAIK: a hash-based algorithm for accurate next-generation sequencing short-read mapping. *PLoS One* **9**: e90581.

Li JW, Wan R, Yu CS, Co NN, Wong N, Chan TF. 2013. ViralFusionSeq: accurately discover viral integration events and reconstruct fusion transcripts at single-base resolution. *Bioinformatics* **29**: 649-651.

MELT. 2016. University of Maryland. Available from: http://melt.igs.umaryland.edu/index.php [Accessed 4 January 2017]

Reuters T. Citation Network. Web of Science. Available from: http://apps.webofknowledge.com [Accessed 4 January 2017]

SourceForge. 2014. Mobster. Available from: https://sourceforge.net/projects/mobster/ [Accessed 4 January 2017]

Sudmant PH, Rausch T, Gardner EJ, Handsaker RE, Abyzov A, Huddleston J, Zhang Y, Ye K, Jun G, Fritz MHY et al. 2015. An integrated map of structural variation in 2,504 human genomes. *Nature* **526**: 75-+.

t-lex. 2011. Stanford University. Available from: http://petrov.stanford.edu/cgi-bin/Tlex.html [Accessed 4 January 2017].

Thung DT, de Ligt J, Vissers LEM, Steehouwer M, Kroon M, de Vries P, Slagboom EP, Ye K, Veltman JA, Hehir-Kwa JY. 2014. Mobster: accurate detection of mobile element insertions in next generation sequencing data. *Genome Biology* **15**.

Tubio JM, Li Y, Ju YS, Martincorena I, Cooke SL, Tojo M, Gundem G, Pipinikas CP, Zamora J, Raine K et al. 2014. Mobile DNA in cancer. Extensive transduction of nonrepetitive DNA mediated by L1 retrotransposition in cancer genomes. *Science* **345**: 1251343.

Wu J, Lee W-P, Ward A, Walker JA, Konkel MK, Batzer MA, Marth GT. 2014. Tangram: a comprehensive toolbox for mobile element insertion detection. *BMC Genomics* **15**: 795.
